# Waterbird Diversity Patterns Under Varied Hydrological Regimes in Dongting Lake and Surrounding Lakes

**DOI:** 10.1002/ece3.72396

**Published:** 2025-10-27

**Authors:** Siqi Zhang, Pingyang Zhang, Yeai Zou, Dongmei Li, Feng Li, Zhengmiao Deng, Jing Zeng, Shengze Wang, Tao Wu, Yucheng Song, Feiyun Li, Wei Xia, Yonghong Xie

**Affiliations:** ^1^ Institute of Subtropical Agriculture Chinese Academy of Sciences Changsha China; ^2^ Technology Innovation Center for Ecological Conservation and Restoration in Dongting Lake Basin Ministry of Natural Resources Changsha China; ^3^ National Field Scientific Observation and Research Station of Dongting Lake Wetland Ecosystem in Hunan Province Changsha China; ^4^ University of Chinese Academy of Sciences Beijing China; ^5^ College of Life Science and Technology Mudanjiang Normal University Mudanjiang China; ^6^ College of Life Sciences Hunan Normal University Changsha China; ^7^ Administrative Bureau of Hunan East Dongting Lake National Nature Reserve Yueyang China; ^8^ Xiangyin Hengling Lake Provincial Nature Reserve Management Committee Yueyang China

**Keywords:** hydrological regime, metacommunity, river‐connected lake, waterbird, waterbird‐habitat network

## Abstract

Globally, climate and human‐induced environmental changes affect the populations and distributions of most organisms. This is particularly true for migratory birds, which change habitats throughout the annual cycle. Understanding how waterbirds adapt to environmental change is important for their conservation; however, this subject remains largely unexplored. Based on wintering waterbirds and environmental data from Dongting Lake and its surrounding lakes, we compared the changes in waterbird diversity in subregions with different habitat variations during four hydrological periods. This allowed us to identify key environmental factors affecting waterbird diversity and explore the potential mechanisms by which waterbirds adjust to these environmental changes using the waterbird‐habitat patch network method. The results indicated that waterbird distribution differed across Dongting Lake and the surrounding wetlands under varying hydrological conditions associated with habitat changes. Notably, the surrounding lakes with stable habitats served as refuges for waterbirds during extreme drought, with the percentage of waterbirds increasing from 9.3% (late recession) to 63.8% (extreme drought). Areas of habitat (i.e., water, mudflat, and vegetation) and human disturbance were key factors influencing waterbird diversity. As indicated by the *β*‐diversity of the waterbird‐habitat patch network, the waterbird metacommunity remained relatively stable in terms of spatiotemporal differences across hydrological periods, while its components displayed distinct patterns. Specifically, the colonization component dominated during extreme drought, whereas the extinction component dominated during the late recession. Compared to normal recession, an earlier recession led to a higher proportion of colonization components. Waterbirds migrated between habitat patches to adapt to the various hydrological conditions. This study enriches the theory of community assembly by emphasizing the significance of extinction and colonization processes shaped by hydrological conditions. Furthermore, it provides valuable insights into the conservation and management of waterbird habitats, highlighting the importance of hydrological dynamics and their impact on waterbird populations.

## Graphical abstract

Extinction‐colonization process explained changes in waterbird diversity patterns. The waterbird‐habitat network method was used to study waterbird diversity patterns. Important and high variability habitat patches were identified. The total diversity of waterbirds in the Dongting Lake region was relatively stable. Waterbirds relocated as the habitat changed in the Dongting Lake region.

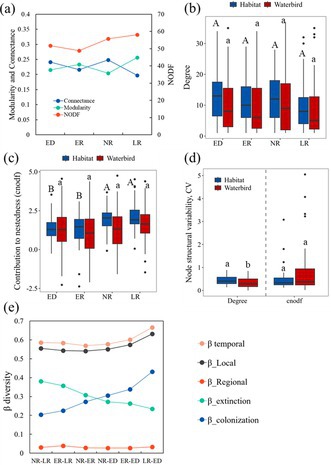

## 1 Introduction

Waterbird diversity is one of the most critical biodiversity factors influencing wetland ecosystem functions, such as nutrient cycles and energy flow processes (Green and Elmberg 2014; Qiu et al. 2024). Waterbirds are highly dependent on wetlands for most or all of their life cycles and are sensitive to habitat changes (Haig et al. 1998). However, ongoing climate change and human activities have drastically altered the distribution, number, and natural dynamics of wetlands, posing significant challenges to the maintenance of waterbird diversity (Lu et al. 2024; Murray et al. 2022; Yang et al. 2024). Understanding the spatiotemporal distribution variation of waterbirds and their shaping factors is important for the conservation and habitat management of waterbirds.

Multiple factors, including the hydrological regime, habitat composition and area, human disturbance, and conservation efforts, can influence the distribution of waterbirds (Li, Yang, et al. 2019; Malekian et al. 2022; Wang et al. 2023). Among these, the hydrological regime is the most important factor for determining the maintenance of wetland structure and functions (Euliss et al. 2004; Xue et al. 2024), and greatly affects the diversity and distribution of waterbirds (Wu et al. 2021; Zhang et al. 2016). However, in river‐connected lakes, the hydrological regime can dramatically change. These changes lead to significant interannual variations in food and habitat resources within wetlands, affecting the diversity and distribution of waterbirds (Zhang et al. 2016, 2018), and introducing challenges to their survival (Gao et al. 2023; Zhang et al. 2023). In contrast, the isolated lakes surrounding them tend to remain at a stable water level, resulting in stable habitat composition and distribution during the wintering period. However, as part of the waterbird‐habitat network, highly mobile waterbirds migrate among habitat patches; thus, the waterbird diversity in these environmentally stable habitat patches can be influenced by waterbird populations in the surrounding areas (Pearse et al. 2024; Zhang et al. 2024). This implies that, in addition to local environmental factors, regional processes (spatial dynamics linked to the dispersal of waterbirds) also affect the distribution of waterbird diversity, especially in river‐connected lakes with significant changes to hydrological regimes.

Understanding the impacts of hydrological regimes on waterbird communities is crucial for waterbird diversity conservation and wetland management (Li, Li, et al. 2019; Wen et al. 2011). Previous studies have demonstrated that hydrological regimes in river and lake wetlands significantly affect waterbird diversity and community structure. For instance, waterbird functional diversity is reduced in constant high water levels (Cui et al. 2024; Ji et al. 2025), while earlier water recession decreases richness and abundance (Wei and Zhou 2023). Furthermore, previous studies have widely explored the effects of environmental variables and waterbird diversity distribution in lake groups (Gao et al. 2021; Li, Yang, et al. 2019; Wang et al. 2023). However, research remains scarce on how hydrological regimes and environmental variables affect waterbird diversity across lake groups subject to differential hydrological influences. This limits our comprehensive understanding of hydrological impacts on waterbird diversity and constrains the development of targeted conservation strategies.

The shallow lakes distributed in the middle and lower Yangtze River floodplain form a key region for migratory waterbirds as their wintering ground along the East Asian‐Australasian Flyway (EAAF) (Cao et al. 2008, 2010). Stable periodic hydrological rhythms provide abundant food and habitat resources for a diverse range of wintering waterbirds. However, in the context of human disturbance, the construction of the Three Gorges Dam (TGD) has resulted in an abnormal hydrological regime that is becoming increasingly prevalent in this region (Li et al. 2020; Mei et al. 2015). This poses a serious threat to the survival of waterbirds (Wang et al. 2017; Zhao et al. 2012). Dongting Lake is the second largest freshwater lake in China and a Ramsar site along the EAAF (Cao et al. 2008). As one of the 200 key ecological nature reserves in the world, it serves as an important wintering ground for waterbirds (Olson and Dinerstein 2002). As the first river‐connected large lake downstream of the TGD, its hydrological regime has changed conspicuously (Hu et al. 2015). Meanwhile, there are a number of small and medium‐sized isolated lakes surrounding Dongting Lake that exhibit relatively stable water levels and habitat characteristics, and which play an important role in supporting waterbird populations. As habitats change with hydrological variation, waterbird diversity in the surrounding lakes might also be affected. There has been research on the effect of hydrological regimes and environmental factors on the waterbirds (Zhang et al. 2016, 2018; Zou et al. 2019). However, it remains unknown how they affect waterbird diversity across the lake groups subject to differential hydrological influences.

Here, based on waterbird and environmental data collected over four typical hydrological regimes during wintering periods in Dongting Lake and the surrounding lakes, this study focused on the following: (1) changes in waterbird diversity distribution under typical hydrological regimes, (2) the impact of environmental variables on waterbird diversity, and (3) the spatiotemporal dynamics of the waterbird metacommunity under different hydrological regimes as revealed through the waterbird‐habitat patch network approach, as well as the potential processes and mechanisms involved in maintaining community diversity in waterbirds. This research could offer scientific information for understanding the impact of hydrological regime on waterbird diversity and the potential process, and provides valuable insights into the conservation and management of waterbird habitats.

## 2 Methods

### 2.1 Study Area

**FIGURE 1:**
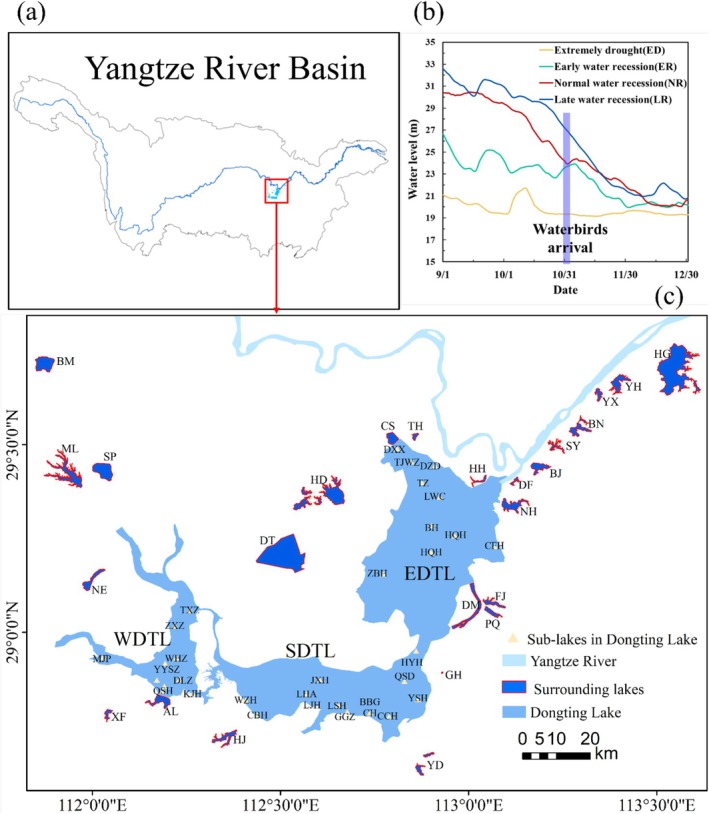
(a) Yangtze River Basin; (b) daily water‐level variations of Dongting Lake during different hydrological periods; (c) study area. EDTL, East Dongting Lake; SDTL, South Dongting Lake; WDTL, West Dongting Lake.

The study area encompasses the Dongting Lake group (including Dongting Lake and 25 surrounding lakes) (Figure 1, Figure S1). The region has a subtropical monsoon climate (Yuan et al. 2015). Dongting Lake (DTL, 28°30′–30°20′ N, 111°40′–113°10′ E) is a typical interconnected river–lake system with a surface area of 2625 km^2^. Water from the Yangtze River enters Dongting Lake primarily through three inlets: Songzi, Taiping, and Ouchi. Additionally, Dongting Lake receives inflows from four major tributaries (Xiangjiang, Zishui, Yuanjiang, and Lishui). Finally, it flows into the Yangtze River at Chenglingji station. As a seasonally inundated water body, its water level fluctuates seasonally and annually, ranging from approximately 36 m in summer to below 20 m in winter. As the water recedes during the dry season, many relatively separated sub‐lakes are formed within Dongting Lake, which refers to relatively isolated areas (Figure S1). Water recession typically occurs in mid‐autumn, and it leads to the gradual emergence of lake shoals and the growth of vegetation that serves as food for waterbirds. The main landscape types of Dongting Lake are water bodies, mudflats, and vegetation. The vegetation is dominated by *Carex*, *Miscanthus*, and *Populus*. Among these, *Carex* meadows are the primary habitat for herbivorous birds, whereas vegetation such as *Miscanthus* and *Populus* is rarely utilized by these birds. Dongting Lake encompasses three Ramsar sites, East Dongting Lake (EDTL), South Dongting Lake (SDTL), and West Dongting Lake (WDTL), which exhibit different hydrological conditions and habitat compositions (Liu et al. 2023). The EDTL is characterized by extensive areas of sedge *Carex*, mudflats, and open water, which are highly influenced by the hydrological regime. In contrast, SDTL and WDTL have larger expanses of *Miscanthus* reeds as well as *Populus* trees, with river channels widely distributed throughout these regions.

The numerous smaller lakes scattered around Dongting Lake also play an important role in the conservation of overwintering waterbirds (Zhang et al. 2024). Most of these lakes resulted from historical lake reclamation and agricultural activities, and they were once part of Dongting Lake. The total area of the surrounding lakes selected for our study is 362.23 km^2^, with 18 protected areas (Table S2). Owing to their isolation from the Yangtze River and the presence of levees or sluices, these lakes experience relatively minor annual and seasonal hydrological changes and maintain relatively stable habitat characteristics.

### 2.2 Waterbird Data

The wintering period of waterbirds in the study area typically lasts from early November to March. Bird survey data were collected in mid‐November from 2019 to 2022. The surveys were conducted simultaneously by multiple groups of trained investigators following a standard protocol. The surveys covered all lakes, although some lakes had missing data during some periods owing to access constraints (Tables S1 and S2). The birds were observed using a 20–60 × spotting scope or 10 × 42 binoculars. In this study, we divided the study area into four groups, EDTL, SDTL, WDTL, and the surrounding lakes (SLS), to explore the effects of hydrological changes on the waterbird metacommunity in the Dongting Lake group.

Regarding the different habitat requirements of the various functional waterbird groups, we considered that the composition of these functional groups could change during different hydrological periods. Waterbird species were classified into nine functional groups according to their diet, habitat preferences, and the manner in which they acquired resources in the wetlands (Almeida et al. 2020; Wu et al. 2014). These groups were similar to those defined by Almeida et al. (2020), although we grouped the gulls together due to the small number of gull species. Considering the large number of geese and cranes in the study area, which were not included in Almeida et al. (2020), we also referred to the study by Wu et al. (2014) and classified them into herbivorous geese and tuber‐feeding birds. The nine functional groups were dabbling ducks, diving ducks, diving fishers, gulls, large and small wading birds, herbivorous geese, tuber‐feeding birds, and vegetation gleaners (Table S3).

### 2.3 Hydrological Regime

To analyze the effects of hydrological regimes on waterbird diversity, the hydrological regimes of four consecutive wintering periods (2019/2020–2022/2023) were classified into four hydrological regimes according to the water recession patterns during the winter period (Figure S2). The annual recession pattern of the lake was obtained based on its average daily water level in October, which is the key month before waterbird arrival (Zhang et al. 2023). A Kruskal‐Wallis test was conducted to compare the October water level in four study years and the 30‐year mean before the TGD (see details in Supporting Information S3). The classifications were as follows: early water recession (ER) for 2019/2020, late water recession (LR) for 2020/2021, normal water recession (NR) for 2021/2022, and extreme drought (ED) for 2022/2023 (P. Zhang, Zou, et al. 2021; Zhang et al. 2023, 2024).

### 2.4 Environmental Factors

Based on expert knowledge and a literature review, 14 environmental variables were selected to analyze their potential influence on waterbird distribution and diversity (Table 1) (Wang et al. 2023; Xu et al. 2024; S. Zhang, Zhang, et al. 2021; Zou et al. 2019). A more detailed description is provided in the Supporting Information (Table S4).

**TABLE 1:** Description of environmental variables.

Classification	Environmental variables	Designated research region
Habitat condition	Area	DTL/SLS
	Water area	DTL/SLS
	Mudflat area	DTL/SLS
	Vegetation area	DTL/SLS
	NDVI	DTL
Local hydrological condition	InDave	DTL
	InD1	DTL/SLS
	InD2	DTL/SLS
Morphological characteristics	Shape index	SLS
MWD	MWD	SLS
Human disturbance	Disturbance	DTL
	Road density	SLS
	Resident density	SLS
Protection	Protection level	SLS

Abbreviations: DTL, Dongting Lake; InD1, inundation duration < 20 days; InD2, inundation duration 20–70 days; InDave, average inundation duration; MWD, mean water depth.

The average inundation duration, inundation duration of less than 20 days (InD1), and inundation duration between 20 and 70 days (InD2) were used to reflect the local hydrological conditions (Table S4, Figure S3) (Teng et al. 2022). It is measured between 1 September and 30 November each year of the study (Zhang et al. 2023). This time frame was selected because the study area is typically flooded before September, and waterbird surveys in the study area are usually completed in mid‐November. The inundation duration of the lake was calculated according to Zhang et al. (2023). The mean water depth (MWD) of the lake was derived from the global lake bathymetry dataset (Khazaei et al. 2022).

The areas of the water, mudflats, and vegetation in the study area were determined using Sentinel‐2 satellite images during the winter periods of 2019/2020–2022/2023 around the time of the survey. The Support Vector Machine technique was employed using the ENVI 5.3 software for the supervised classification of these images. A total of 500 training samples were selected by subsetting the regions of different habitats, which were then compared to 500 samples of known cover (acquired with field surveys and Google Earth maps). *Miscanthus* and *Populus* are seldom utilized by waterbirds; therefore, the vegetation in Dongting Lake includes only the *Carex* area. *Miscanthus* and *Populus* masks were extracted as described by Huang et al. (2022). Regarding the relatively small vegetation areas, specific vegetation categories were not distinguished in the surrounding lakes. The normalized difference vegetation index (NDVI) was extracted from Sentinel‐2 satellite images using the following equation: NDVI = (NIR − R)/(NIR + R), where NIR represents near‐infrared reflectance and R represents visible red light reflectance.

The protection status of these lakes was acquired from the Forestry Department of Hunan Province, and ordinal values were assigned according to the level of protection (Table S2). The areas within Dongting Lake were not considered in terms of protection status because the entire lake is located in a protected area. Residential and road data were obtained from the National Catalogue Service for Geographic Information (https://www.webmap.cn/main.do?method=index). The lake area and perimeter were calculated using ArcGIS 10.2. The shape index was calculated using the following formula (Wang et al. 2023):

$$\mathrm{SI}=L/2\sqrt{\pi \times A}$$

where *L* is the wetland perimeter (km) and *A* is the wetland area (km^2^). The shape index was used to indicate the complexity of lake shapes, with higher values indicating greater complexity.

The residential density and road density were calculated as the number of settlements or road lengths (within a 1.5 km buffer zone around the lake) divided by the area of the lake, using ArcGIS 10.2. Because Dongting Lake is located in a nature reserve where few roads exist, we referred to Zou et al. (2019) and categorized the degree of human disturbance in Dongting Lake as ordinal data according to the intensity of human activity (Table S1).

### 2.5 Statistical Analysis of Data

The abundance (number of individual waterbirds), species richness, Shannon‐Wiener diversity index (SHDI), and Pielou evenness index (Pielou 1966) were calculated to measure the species diversity of the communities. The kernel density estimation of the waterbirds was conducted using ArcGIS 10.2. The cell size was set to 50 m, the search radius to 5 km, and the area unit scale factor to square kilometers.

To detect variance in species and functional group composition across different hydrological periods, principal coordinates of association (PCoA) analyses were performed on the matrices of counts across different periods of EDTL, SDTL, WDTL, and SLS, which were based on species and functional group abundance. A permutational multivariate analysis of variance was used to test for differences in waterbird assemblage composition between different subregions (different wintering periods) at both the subregion and local scales. The R package *vegan* was used (Oksanen et al. 2020), and the Bray‐Curtis distance measure was employed for the PCoA analysis. The redundancy analysis (RDA) of functional group and habitat area (water, mudflat, and vegetation) was implemented.

Therefore, we considered the possibility that the impact of the hydrological regime on habitat composition varied across different subregions. Distance‐based permutational tests on multivariate dispersion (PERMDISP) (Anderson et al. 2006) were used to detect the extent of variation in habitat composition during different hydrological periods in the four subregions. The PERMDISP method compares the distances of points within each group (subregion) to their central points, thereby testing the extent to which the habitat composition in different regions is affected by hydrological changes. Following PERMDISP, Kruskal–Wallis tests were used to further examine differences in habitat area and local hydrological changes under different hydrological regimes.

Partial least squares structural equation modelling (PLS‐SEM) is used to estimate the causal network among latent variables, and the latent variables can be expressed by a set of manifest variables. It is a multivariate statistical analysis method used to establish or verify the multipath relationships step by step among multiple independent variables and dependent variables based on a limited dataset. PLS‐SEM relaxes the assumption of multivariate normal distribution in the parameter estimation process (Shen et al. 2016), and it is more suitable for exploratory research on small samples and can effectively evaluate the interaction between variables (Fan et al. 2016; Tenenhaus et al. 2005). The diversity of waterbirds in the lakes is influenced by different factors directly or indirectly, which is difficult for normal statistical analysis methods. In this study, PLS‐SEM was used to evaluate the essential factors affecting the waterbird diversity. The analysis was performed within R using the “plspm” package (Sanchez et al. 2015).

Multiple linear regression was employed to explore the main environmental factors affecting the diversity indices of the waterbird assemblages. The VIF (Variance Inflation Factor) function from the *car* package (Fox et al. 2024) was applied to check for multicollinearity among the model variables. A VIF value greater than 10 is considered to indicate high multicollinearity (Dormann et al. 2013; Gao et al. 2021). After considering the ecological significance and importance, variables with high VIF values and lower importance were removed from the model. Then recheck the VIF values of the remaining variables in the model until the VIF values of all selected variables are no greater than 10 (Tables S5 and S6). Afterward, the optimal subset regression was used to select the subset of variables based on the lowest Bayesian information criterion value using the R package *leaps* (Miller 2024), which can perform an exhaustive search to select the model. Subsequently, a multiple linear regression was used to fit the selected variables. Part of the data was transformed using the log (*x* + 1) method for the analysis of the normalized data (Figure 6). The coefficients of the variables were standardized using the *lm.beta* package (Behrendt 2023) in R (R Core Team 2024).

The changes in the waterbird‐habitat patch network within the study area under different hydrological periods were calculated following the methods of Li et al. (2023). This species‐patch network approach quantifies temporal changes (*β* temporal) in network structure and the temporal beta diversity of species‐patch links and its components, reflecting species extinction‐colonization and patch gain and loss, providing insights into the metacommunity assembly process. The metacommunity is treated as a species‐patch network with species and patches as nodes, species occurrences as links, such a network approach explicitly connects multiple species and patches. Then, the temporal beta diversity of species‐patch links between different time points is used to quantify temporal metacommunity dynamics. The four distinct hydrological periods were treated as separate time points, and the differences in waterbird‐patch networks were calculated between each pair of time points. The *Jaccard* dissimilarity is used to calculate the beta diversity of networks. The temporal beta diversity (*β*_temporal_) of species‐patch links at two time points can be further partitioned into four components:

$$\beta_{\text{Temporal}}=\beta_{\text{Local}}+\beta_{\text{Regional}}+\beta_{\text{Landscape}}+\beta_{\mathrm{RL}}$$

where *β*_Temporal_ represents temporal beta diversity, *β*_Local_ represents local component, *β*_Regional_ represents regional component, *β*_Landscape_ represents landscape component, *β*_RL_ represents regional landscape component (Table S7).

Since the difference in links between two networks from two time points was involved in extinction–colonization dynamics, the temporal beta diversity of species‐patch links can be directly partitioned into extinction‐ and colonization‐driven components:

$$\beta_{\text{Temporal}}=\beta_{\text{Extinction}}+\beta_{\text{Colonization}}$$

where *β*_Temporal_ represents temporal beta diversity, *β*_Extinction_ represents extinction component, *β*_Colonization_ represents colonization component (Table S7).

The detailed information is available at Li et al. (2023). An overview of the components of temporal dissimilarity (beta diversity) of the species‐patch networks is shown in Table S7. These indices were calculated in R using the code provided by that study. Network‐level metrics, including the connectance, nestedness, and modularity indices, were calculated to depict the properties of the waterbird‐habitat patch network, as suggested by Li et al. (2023). Node‐level changes were assessed using the degree and contribution of particular nodes to the network structure (the nestedness contribution [cnodf]) and their coefficient of variation (CV). The degree describes the number of partners in a given node. The degree of a patch is the richness of the patch, whereas the degree of waterbirds describes the width of the spatial niche of the waterbirds, which is measured as the number of patches occupied by waterbirds. The cnodf is used to identify keystone patches that can disproportionately affect the dynamics of the metacommunity. The CV was used to test the temporal variability of the nodes (stability of the patches). Important sites with higher stability in the habitat network were identified based on the cnodf and CV indices of each node. Detailed calculation information is provided in Supporting Information S9. These indices were calculated using the *bipartite* package (Dormann et al. 2008) in R. For data integrity, the analysis excluded site data with missing values across the four typical hydrological periods.

For data that exhibited abnormal distributions, as determined by the Shapiro–Wilk test, the Kruskal–Wallis test was used to examine whether the degree and cnodf differed significantly among the four hydrological periods. The Mann–Whitney *U* test was used to examine whether the CV of degree and the cnodf significantly differed between waterbird and habitat patches. The significance level was set at *p* < 0.05.

## 3 Results

### 3.1 Waterbird Diversity Patterns in Dongting Lake and Surrounding Lakes

**FIGURE 2:**
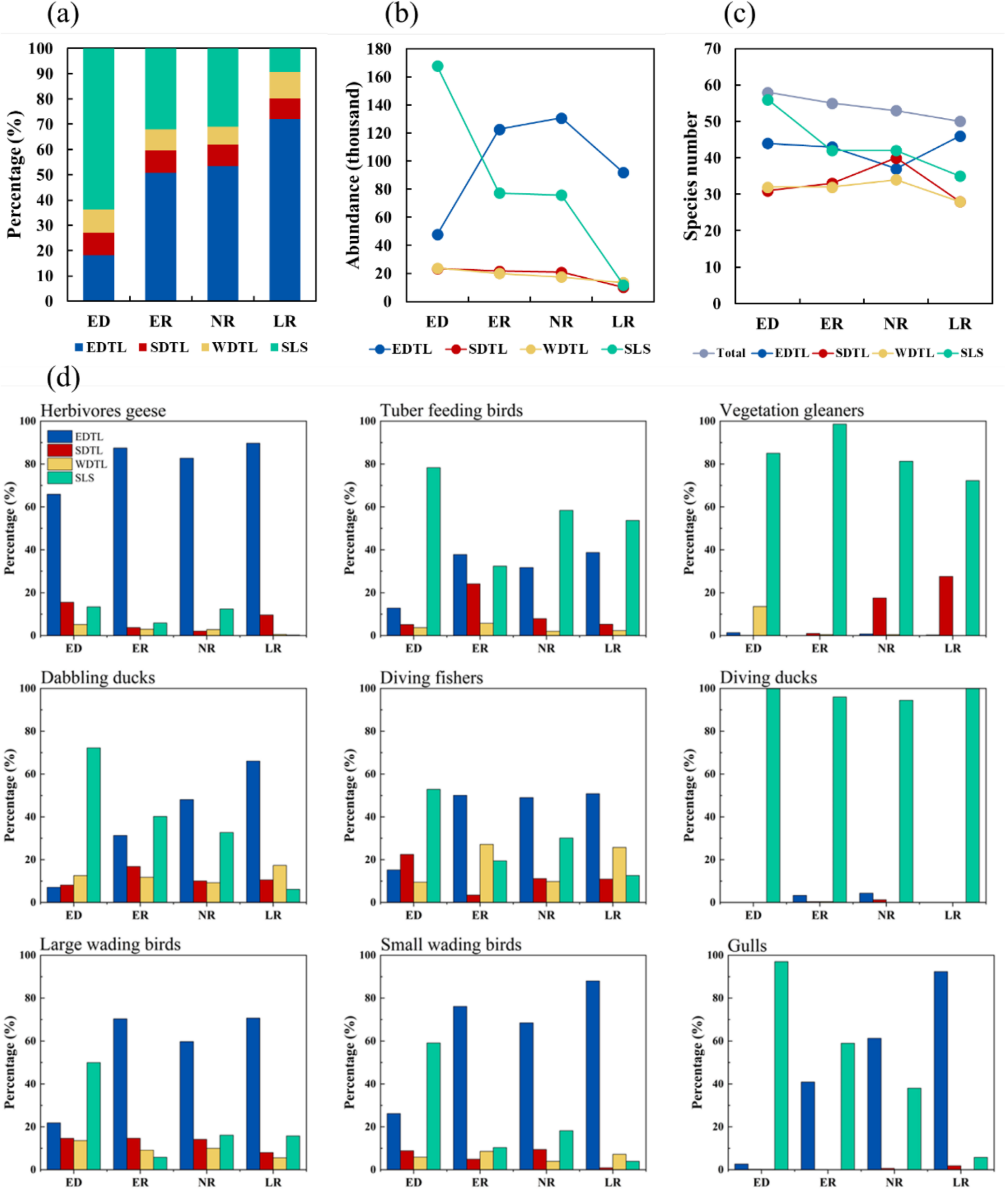
Waterbird distribution in subregions across Dongting Lake and the surrounding lakes. (a) Percentage of waterbird abundance during different hydrology periods. (b) Waterbird abundance during different hydrology periods. (c) Species number (richness) of waterbirds. (d) Percentage of functional group of waterbirds during different hydrological regimes. ED, extreme drought; ER, early recession; LR, late recession; NR, normal recession.

**FIGURE 3:**
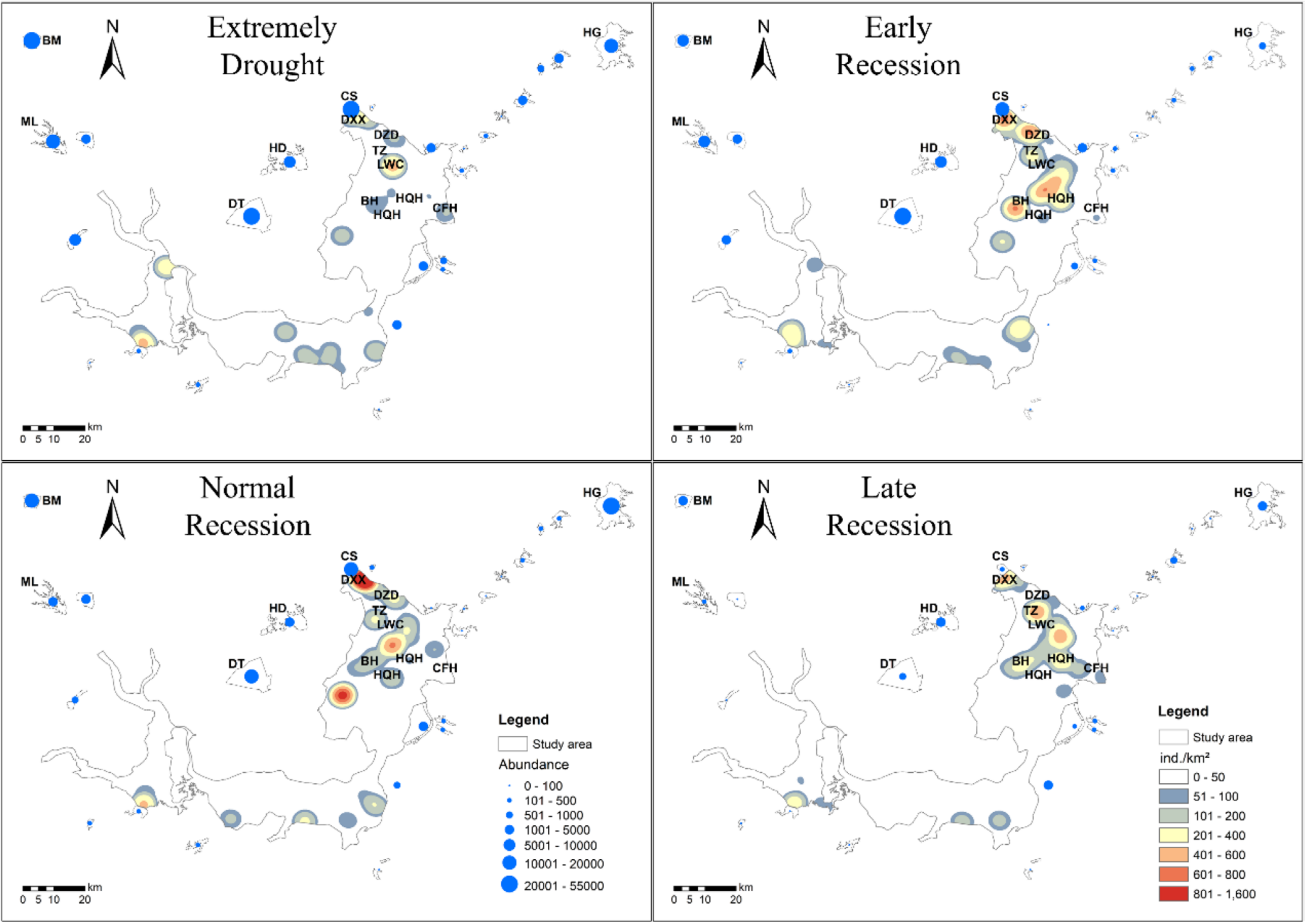
Distribution of waterbirds in the study area during different hydrology periods. The kernel density estimation of waterbird abundance in Dongting Lake (DTL); the abundance of waterbirds is represented by the size of circles in surrounding lakes (SLS).

A total of 876,959 waterbirds belonging to 68 species were recorded during the 2019/2020–2022/2023 wintering periods (Table S3). Although the total number of species was relatively stable over the four study periods, regional variations were evident. The total species number was higher in EDTL and SLS, but lower in SDTL and WDTL (Figure 2c). The distribution patterns of waterbirds in the EDTL and SLS exhibited distinct characteristics across different hydrological periods. In contrast, the SDTL and WDTL remained relatively stable (Figures 2 and 3). Most waterbirds were concentrated in the EDTL, except during periods of extreme drought, when their numbers significantly decreased in the EDTL but increased in the SLS (Figures 2 and 3; Tables S8–S11). As for different functional groups, water‐dependent waterbird groups in EDTL (e.g., dabbling ducks, diving fishers, wading birds, and gulls) have shown a trend of migration to SLS during the extreme drought. However, the geese remain predominantly distributed within EDTL during the extreme drought period (Figure 2d).

Within the EDTL (during the ER, NR, and LR periods), waterbirds were primarily distributed in areas such as BH (21.5% on average), DXX (12.7% on average), TZ (5.7% on average), HQH (5.5% on average), and DZD (5.3% on average), which offered diverse and rich habitats, as reflected by their diversity indices (Figure 3, Figures S1 and S4–S6). During extreme drought, waterbirds in EDTL were concentrated in LWC (4.9%), DZD (3.7%), and DXX (3.3%) (Figure 3, Figures S1 and S4–S6). Moreover, the average abundance of waterbirds in the EDTL drastically decreased during extreme drought compared to that in other hydrological regimes (Figures 2 and 3, Figure S1, Table S8). As for the surrounding lakes, waterbirds were primarily concentrated in the larger lakes: DT (10.0% on average), CS (6.1% on average), HG (4.3% on average), BM (4.1% on average), ML (2.1% on average), and HD (1.9% on average); these areas exhibited above‐average abundance and species richness (Figure 3, Figures S4 and S7). Notably, the average diversity indices, such as abundance, richness, and SHDI, increased during extreme drought in the SLS (Tables S8–S10).

**FIGURE 4:**
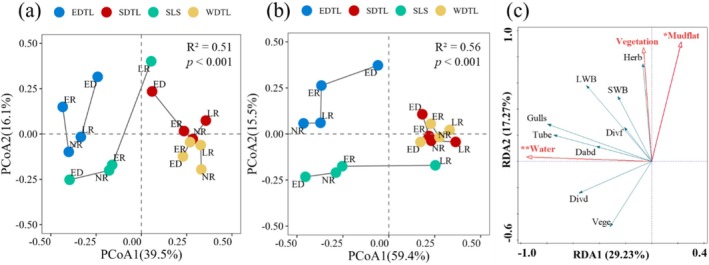
PCoA ordination map based on the Bray‐Curtis distance of species and functional group abundance with the result of the permutational multivariate analysis of variance test. (a) Species abundance data; (b) functional group data. (c) RDA analysis between habitat and functional groups (*p < 0.05, **p < 0.01, first axis: *F* = 5, *p* < 0.05; all axis: *F* = 3.7, *p* < 0.01). The functional groups are shown in the figure. Dabd, dabbling ducks; Divd, diving ducks; Divf, diving fishers; Herb, herbivorous geese; LWB, large wading birds; SWB, small wading birds; Tube, tuber feeding birds; Vege, vegetation gleaners.

The compositional dissimilarity of waterbird communities varied significantly across the subregions (species composition, *F*_3,12_ = 4.16, *p* < 0.001; functional group composition, *F*_3,12_ = 5.08, *p* < 0.001; Figure 4). Waterbird communities in the EDTL and SLS sites were distinct from those in the SDTL and WDTL sites. The species and functional group composition of waterbirds in EDTL and SLS exhibited substantial changes under different hydrological regimes, whereas SDTL and WDTL showed relatively stable compositions (Figure 4, Figure S8).

At the local scale, the waterbird communities in EDTL were significantly different from those in the other three subregions during the four hydrological periods (Figure S9, Table S12). Within the subregions of DTL, the waterbird community composition at the local scale did not differ significantly between hydrological periods, indicating the redistribution of waterbirds within the subregions (Figure S10, Table S13).

### 3.2 Driving Factors of Waterbird Diversity Changes in Dongting Lake and Surrounding Lakes

The hydrological regime emerged as the primary factor driving changes in waterbird diversity in DTL and SLS (Figures 2, 3, 4). Extreme drought conditions significantly altered habitat and reduced the suitable area for waterbirds in the EDTL (Figures S11 and S12), which resulted in a notable decline in waterbird abundance (Figure 2). Conversely, in SLS, where the hydrological conditions were relatively stable (Figures S11 and S12), waterbird diversity increased during extreme drought because these lakes served as important refuges for waterbirds displaced from the EDTL.

The area of different habitat types was significantly correlated with functional group composition (Figure 4c), indicating that variations in functional group composition were linked to habitat differences and changes. Furthermore, the functional groups of waterbirds were more sensitive to habitat changes. The habitat composition variation in EDTL was significantly higher than that in the other three subregions and was significantly higher than that of WDTL and SLS, while SLS showed the lowest variability (*F*_3,12_ = 24.12, *p* < 0.001; Figure S11). Moreover, the habitat area in EDTL also showed considerable variability across the hydrological regimes, whereas those in SDTL and WDTL remained relatively stable (Figure S12). The habitat area in the SLS was the most stable and unaffected by the hydrological regimes in the DTL (Figures S11 and S12). The observed functional group variations in EDTL were primarily driven by changes in the habitat area under different hydrological regimes. In contrast, the shift in SLS was likely influenced by other factors, such as waterbird movement from the DTL due to hydrological changes, as well as variations in interannual waterbird arrivals.

The PLS‐SEM revealed the influence pathways of various environmental factors on waterbird diversity. The results show that hydrological regime has a significant effect on habitat area (*β* = −0.04, *p* < 0.01) and NDVI (*β* = 0.56, *p* < 0.001) in DTL. The wetland area (*β* = 0.69, *p* < 0.001) and local hydrological condition (*β* = 0.32, *p* < 0.01) had a significant positive effect on habitat area. The waterbird diversity was significantly affected by habitat area (*β* = 0.65, *p* < 0.01) and disturbance (*β* = −0.20, *p* < 0.05). Habitat area (0.65) has the greatest impact on diversity, followed by wetland area (0.45), disturbance (−0.20), NDVI (0.15), hydrological regime (0.09), and local hydrological condition (0.06). As for the SLS, wetland area (*β* = 0.56, *p* < 0.001) and local hydrological condition (*β* = 0.46, *p* < 0.001) have a significant positive effect on habitat area. The habitat area (*β* = 0.75, *p* < 0.001) and disturbance (*β* = −0.22, *p* < 0.05) have a significant effect on diversity. Similar to that within Dongting Lake, habitat area (0.75) has the greatest impact on diversity, followed by wetland area (0.42), disturbance (−0.22), waterbird abundance in DTL (−0.14), local hydrological condition (0.06), mean water depth (−0.06), protection (0.05), and SI (−0.003). Wetland area plays an important role in determining habitat area. The hydrological regime in Dongting Lake affects waterbird diversity by altering the area of different types of habitats.

After the check of the VIF, the total area of wetland in DTL and SLS was removed (Tables S5 and S6). Environmental variables played crucial roles in shaping the spatial distribution of waterbird diversity in DTL (abundance: *F*_2,106_ = 12.63, *p* < 0.001; richness: *F*_2,106_ = 20.70, *p* < 0.001; SHDI: *F*_1,107_ = 13.74, *p* < 0.001; Pielou: *F*_2,105_ = 1.50, *p* > 0.05, Figure 6) and SLS (abundance: *F*_3,92_ = 36.57, *p* < 0.001; richness: *F*_3,92_ = 32.79, *p* < 0.001; SHDI: *F*_4,91_ = 15.83, *p* < 0.001; Pielou: *F*_1,87_ = 6.50, *p* < 0.05, Figure 6). In the DTL, water and vegetation areas in sub‐lakes positively influenced waterbird abundance, whereas the area of InD2 had a positive effect on richness and SHDI. Conversely, human disturbance negatively affected waterbird richness. In the SLS, water and mudflat areas positively affected waterbird diversity metrics, such as SHDI, richness, and abundance. However, the vegetation area and RED (a proxy for human disturbance) had negative effects.

### 3.3 Potential Process of Spatial–Temporal Changes in Waterbird Diversity

Waterbirds exhibit remarkable adaptability to changing habitat conditions by altering their distribution patterns and utilizing different habitat patches. Analysis of the waterbird‐habitat patch network provided valuable insights into the regional process of changing the diversity distribution, as well as the mechanisms maintaining waterbird diversity. Across different hydrological periods, the network‐level metrics showed only slight variations (Figure 7a–c). However, the CV of habitat degree was significantly higher than that of waterbirds (*Z* = 2.66, *p* < 0.01), while the CV of waterbird cnodf was higher than that of habitats, though not significantly different (*Z* = −0.64, *p* = 0.95). This suggests that the temporal structural variability of habitat patches is greater than that of waterbirds (Figure 7d).

The *β*_temporal_ values between pairs of hydrology periods remained relatively stable, driven primarily by local components rather than regional ones (Figure 7e). The extinction–colonization dynamics within the network varied significantly across the hydrological periods. During extreme drought, colonization emerged as the dominant process, whereas during the late recession periods, extinction became predominant (Figure 7e). Compared to normal recession periods, earlier recession periods saw an increase in colonization, which became the dominant component during extreme drought (Figure 7e). These findings suggest that waterbirds actively adjust their distribution in response to habitat changes caused by varying hydrological conditions, helping to maintain the diversity of metacommunities within the region.

Based on the index of cnodf and its CV, the observed stability and importance of certain habitat patches were identified, providing key insights into the process of waterbird diversity changes during different hydrological periods (Figure 8, Supporting Information S9). Habitat patches that could disproportionately affect the dynamics of the metacommunity were identified, with 11 of these patches located within the DTL (EDTL: six patches, SDTL: three patches, WDTL: two patches), and ten patches located in the SLS. Patches with higher temporal structural variability were mainly distributed in the EDTL (EDTL, three patches; SDTL, two patches; SLS, one patch). A higher temporal structural variability of patches could be associated with a higher level of habitat changes and the extinction‐colonization process. These patches may require more attention under changing hydrological regimes. The identified patches could provide important insights for conservation and management strategies (Figure 8, Figure S13).

## 4 Discussion

**FIGURE 5:**
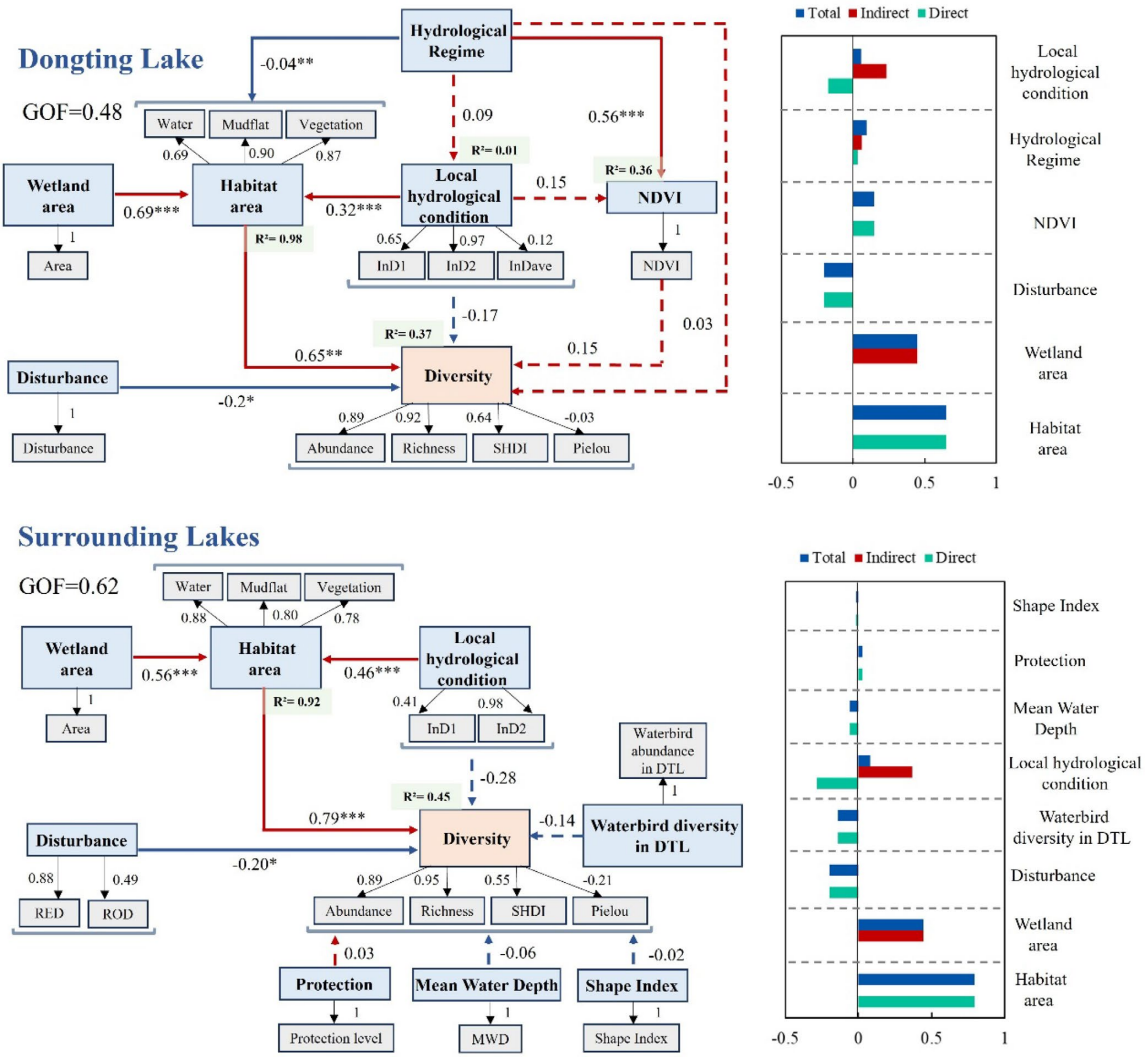
PLS‐SEM path coefficients and significance testing results of the structural model (*p < 0.05, **p < 0.01, ***p < 0.001).

**FIGURE 6:**
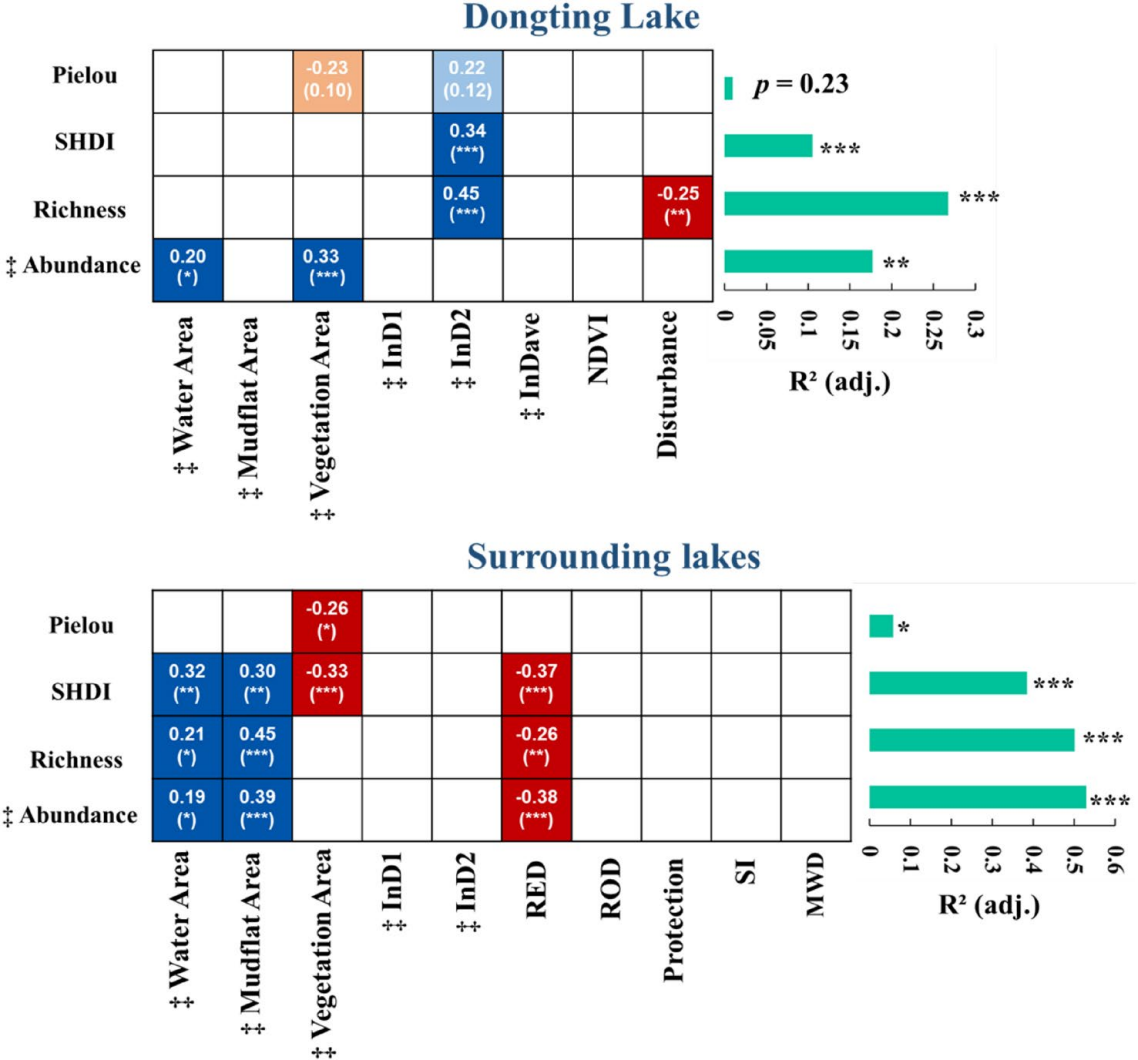
Relationship between the diversity indices of waterbirds and environmental variables. Blue indicates a significant positive effect, red indicates a significant negative effect, light blue and orange indicate insignificant positive and negative effects. The values in the grid represent the standardized coefficients. *p < 0.05, **p < 0.01, ***p < 0.001. ^‡^The variable was log (*x* + 1) transformed. One was added to the abundances before log‐transforming to avoid issues with zero. Variables with VIF > 10 were removed. MWD, mean water depth; RED, resident density; ROD, road density; SI, shape index.

We examined the spatial and temporal patterns of waterbird diversity over four consecutive wintering periods. These periods represent four typical hydrological regimes in the large river‐connected Dongting Lake and its surrounding lakes. Our study revealed that the hydrological regime significantly affected the distribution of waterbird assemblages; this distribution change was associated with habitat changes. Specifically, waterbird diversity varied across different hydrological periods in EDTL, whereas SDTL and WDTL showed more stability in both the abundance and composition of waterbirds, consistent with habitat variation. Extreme drought lowered the abundance of waterbirds in the EDTL, whereas their abundance increased dramatically in the SLS. The SLS serves as a refuge for waterbirds during extreme drought periods. In addition, the area of habitats (water, mudflats, and vegetation) and human disturbance were key factors affecting the diversity indices of waterbirds (Figures 5 and 6). At the regional scale, waterbirds altered their distribution areas in response to habitat changes caused by varying hydrological conditions, thereby maintaining the diversity of waterbird metacommunities within the region.

### 4.1 Effects of the Hydrology Regime and Waterbird Distribution Dynamics

Hydrological regimes primarily affect the distribution and diversity of waterbirds by influencing habitat and food resource availability (Aharon‐Rotman et al. 2017; Ma et al. 2010; Zhang et al. 2020). As the water recedes, the areas of mudflats and meadows expand; however, late recession delays and reduces their exposure. Decreased areas of mudflats and vegetation negatively affect the numbers and distribution ranges of waterbirds that utilize these habitats (e.g., geese and wading birds) (Cui et al. 2024; S. Zhang, Zhang, et al. 2021). Moreover, early water recession affects habitats by reducing food quality and availability (Gao et al. 2023; Ma et al. 2010; Zhang et al. 2020). A longer vegetation growth period leads to increased plant height and a decline in nutritional value, exacerbating food shortages for herbivorous birds during midwinter (Wang et al. 2013; P. Zhang, Zou, et al. 2021). Also, moist mudflats can dry out after a while, thereby reducing food availability for small wading birds (Aharon‐Rotman et al. 2017). During the extreme drought period, the water area in DTL decreased significantly (Figure S12), which exacerbated these issues and led to a large‐scale disappearance of waterbird habitats, resulting in decreased waterbird abundance.

With annual periodic hydrological rhythms, large areas of water bodies, mudflats, and sedge meadows in EDTL are exposed during the wintering period, providing a rich habitat and food source for various groups of waterbirds (Figure 2d, Figures S8 and S12). This makes EDTL the most important wintering site, and it normally harbors the highest number of waterbirds in the Dongting Lake region (Cao et al. 2008; Zou et al. 2019). Additionally, the composition of waterbird habitats in the EDTL was the most affected by changes in hydrological conditions (Figures S11 and S12), which likely explains why waterbird diversity in the EDTL was more sensitive to different hydrological regimes than the other regions. In contrast, the habitats of SLS were not affected by hydrological conditions, and their area of habitats was stable (Figures S11 and S12). However, the high mobility of waterbirds means that waterbird diversity in a particular area is also influenced by fluctuations in waterbird abundance in surrounding areas (Wang et al. 2019). Interannual variation in waterbird diversity in the SLS was mainly influenced by changes in waterbird abundance in the DTL. During the extreme drought period, the water surface almost completely disappeared in the perennial water areas, with only the channel displaying noticeable water coverage. Consequently, most water‐dependent waterbirds (e.g., tuber‐feeding birds, dabbling ducks, diving fishers, wading birds, and gulls) in the EDTL transferred to the SLS (Figure 3, Figure S7; e.g., DT, CS, and BM lakes), which became refuges for wintering waterbirds (Figure 2d). Previous studies indicated that wetlands with stable habitats can serve as refugia and alternative habitats for waterbirds (Sebastián‐González et al. 2010; Wen et al. 2016). However, geese depend on meadows and exhibit a different adaptation strategy. Even under extreme drought conditions with declining food quality, geese remain predominantly distributed within EDTL (Figure 2d). This may be due to the lack of suitable habitats for geese outside the DTL. Secondly, during the extreme drought conditions, geese grub for the underground plant parts rather than *Carex* leaves (Zhang et al. 2024). It demonstrates their dietary plasticity in coping with decreased food availability. This reflects the divergent adaptive strategies exhibited by different waterbird groups in response to environmental changes. In SDTL and WDTL, large areas of reeds, along with many river channels, were unsuitable for waterbird habitats, resulting in a relatively low number of waterbirds in these areas. Compared to EDTL, habitat composition in SDTL and WDTL was less affected by hydrological regimes. The relatively low number of waterbirds and smaller changes in habitat composition could account for the lower variation in waterbird diversity.

This study indicates that hydrological conditions affect the distribution of waterbirds by affecting their habitat conditions (Figures 4c and 5). However, factors not mentioned here also constrain the diversity of waterbirds, such as the arrival time of wintering waterbirds, interannual fluctuations in waterbird abundance, climatic conditions, and biological interactions (Eyster et al. 2022; Kumar et al. 2024; Zhang et al. 2016). These factors should be incorporated in future studies for a more comprehensive analysis.

### 4.2 Effects of Environmental Factors on Waterbird Diversity

Habitat area is an important factor affecting waterbird diversity (Hamza et al. 2024). Results indicate that wetland area significantly affects habitat area, thereby exerting a substantial impact on waterbird diversity (Figure 5). Hydrological regimes influence waterbird diversity by altering habitat composition (i.e., the area of different habitat types, Figure 5, Figure S12). Notably, different waterbird functional groups utilize and prefer distinct habitat types. The changes in habitat composition and area can affect waterbird structure and diversity (Li, Li, et al. 2019). Further analysis reveals that the key influencing factors vary across different diversity indices. The areas of open water and vegetation are important variables affecting waterbird abundance in the DTL. A larger water area could support a higher abundance of waterbirds, which is consistent with previous findings (Xia et al. 2016). Moreover, herbivorous geese are abundant in the DTL, as they are drawn to the sedge meadows (Zou et al. 2019). The areas of water and mudflats were even better indicators, showing a positive association with abundance, richness, and SHDI, whereas the vegetation area has a negative effect on SLS (Figure 6). It is not surprising that, apart from meadows, there are other types of vegetation in the SLS, such as poplars and reeds. A large area of such vegetation is not conducive to the diversity of waterbirds (Hamza et al. 2024) and negatively impacts SHDI and evenness. Moreover, the presence of mudflats, especially those with large areas, can provide a habitat for a greater variety of waterbird functional groups (e.g., wading birds), which positively contributes to the abundance, richness, and SHDI of waterbirds. Previous studies have indicated that inundation area is a key determinant of waterbird diversity (Jia et al. 2018). The InD2 reflects various habitats (e.g., vegetation and mudflats) in Dongting Lake (Figure S3) which could support many waterbird species and thus showed a positive effect on the richness and SHDI of waterbirds (Figure 6). Inundation areas (reflected by InD1 and InD2) are also crucial for the SLS, leading to the development of meadows and exposed mudflats.

Human disturbance can adversely affect the diversity patterns of waterbirds; it typically leads to biodiversity loss in terms of decreased abundance and richness or increased community similarity (Gavioli et al. 2019; O'Connell et al. 2007; Schuh and Guadagnin 2018). As Dongting Lake is an important protected area and “10‐year Fishing Ban in the Yangtze River”, the human disturbance in DTL was relatively low. However, the human disturbance in SLS is complex, such as agricultural practices (such as fish farming, fishing, aquaculture, and crop cultivation), as well as high‐intensity disturbance resulting from urbanization. These intensive human activities can significantly affect the distribution and abundance of waterbirds (Xu et al. 2022). Furthermore, human disturbance reduces the probability of disturbance‐sensitive species, resulting in a higher occurrence of disturbance‐tolerant species, which negatively affects waterbird diversity (Quan et al. 2002; Wang et al. 2023). However, only limited indices were selected to reflect the human disturbance. These variables could overall reflect the potential extent of human utilization and impacts on wetlands. Nevertheless, it is not identified which specific type of human disturbance has the greatest effect on waterbird diversity. To better understand the impacts of human activities on waterbird diversity and develop more effective conservation strategies, future studies should further differentiate the effects of various types of human disturbance.

### 4.3 Dynamics and Diversity Maintenance of the Waterbird Metacommunity

**FIGURE 7:**
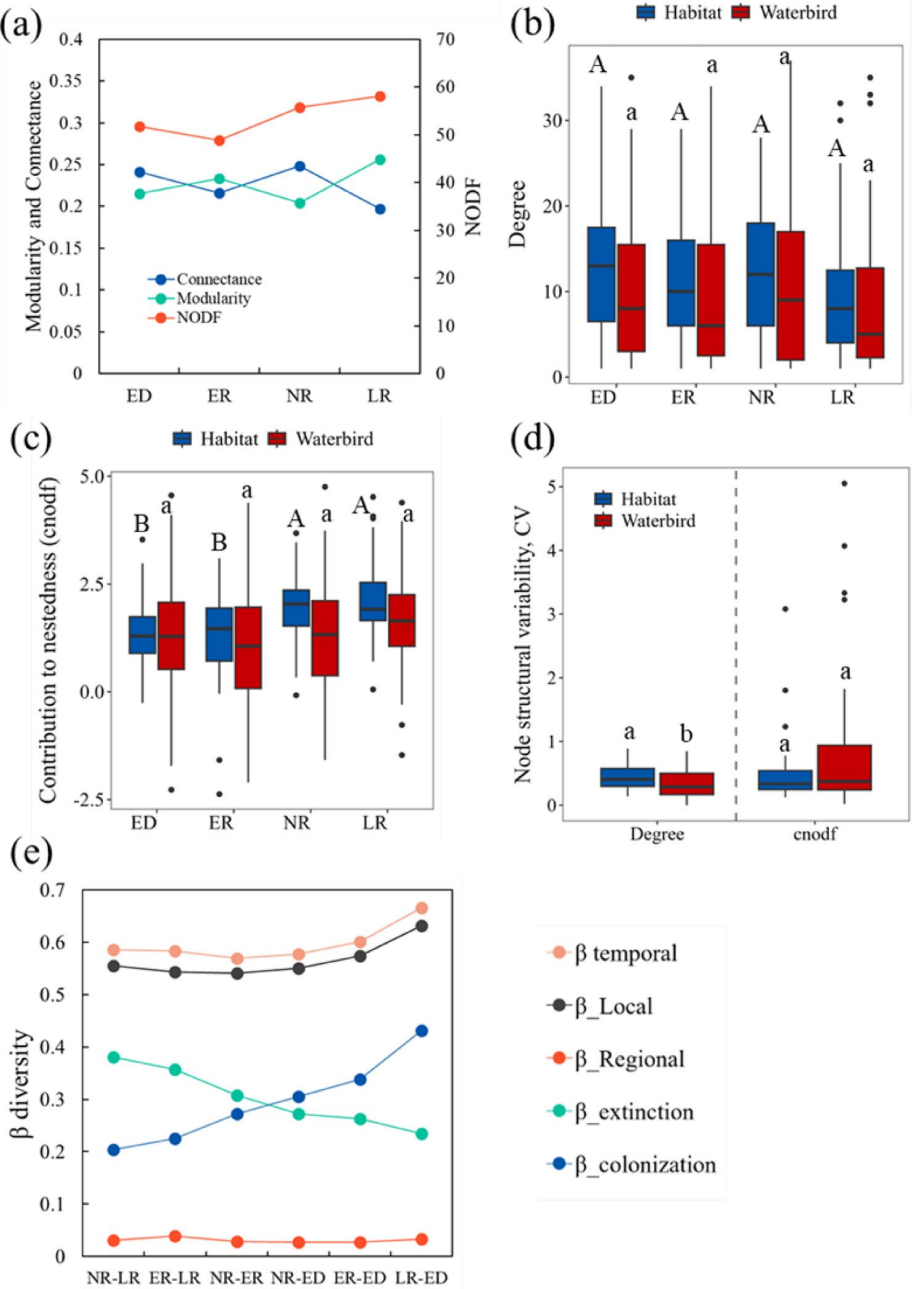
The temporal changes of the waterbird metacommunity. (a) The network metrics of the waterbird‐habitat patch network over different hydrology periods. (b) Degree changes over different hydrology periods. (c) Changes in contribution to nestedness over time. (d) The individual node temporal structural variability of habitat patches and waterbirds in the waterbird‐habitat network. (e) The temporal beta diversity of links between the different hydrology periods.

Waterbirds are highly mobile and sensitive to habitat changes caused by water‐level fluctuations (Wei and Zhou 2023; Zhang et al. 2020). They change their habitat patches in response to resource fluctuations and survival requirements (Zhang et al. 2016). In this study, the spatial niche breadth of specific waterbird species remained relatively stable, whereas the richness of habitat patches fluctuated greatly, which could be attributed to habitat changes within the patches under varied hydrological conditions (Figure 7d). The results showed that, compared with other hydrological conditions, the extinction component during the late recession period dominated the metacommunity network, whereas during the extreme drought period, colonization was dominant (Figure 7e). This was consistent with the changes in the area of habitat within the waterbird patches under varying hydrological conditions (Figures S11 and S12). The waterbirds tended to concentrate in some exposed habitat patches, whereas suitable habitats in other patches remained unexposed during the late recession period (Figures S11 and S12). For instance, wading birds and geese that utilize mudflats and sedge meadows may concentrate on a small number of exposed mudflats and meadow habitats, leading to their disappearance from unexposed habitat patches. Moreover, drought affects the food quality and habitat conditions of waterbirds and has changed their distribution (Gao et al. 2023; Wen et al. 2016; Zhang et al. 2020). During extreme drought periods, the area of habitat for waterbirds decreased drastically and became dispersed in patches, owing to the disappearance of a large area of the water surface. This was particularly true for the EDTL, which could not support a large number of waterbirds as in previous years, leading to the dispersion of waterbirds to SLS. This dispersion resulted in the highest colonization of waterbirds during this period compared to other hydrological conditions.

Studies have suggested that environmental factors predominantly influence bird communities at scales of 1000 km or less, whereas dispersal limitations become more important at larger scales (Meynard and Quinn 2008). The primary process in the assembly of waterbird communities is environmental filtering at small spatial scales, in which the abundance and richness of waterbirds are determined by the quantity and availability of food and habitats (Bonthoux and Balent 2015; Chen et al. 2019; Henry and Cumming 2016; Li, Zhang, et al. 2019). Habitat changes within patches and the high dispersal ability of waterbirds jointly drive changes in the distribution of waterbirds within the metacommunity. Moreover, many wintering waterbirds exhibit strong site fidelity, which may reduce movement among lakes and increase the similarity of communities at the same site across different hydrological periods (Blums et al. 2002; Chan et al. 2023). Therefore, in the context of drastic interannual hydrological changes in river‐connected lakes, waterbirds are likely to initially shift their habitats within a small range, such as by transferring between adjacent sub‐lakes within Dongting Lake, when faced with relatively minor changes in hydrological conditions and habitats (Wei and Zhou 2023). However, under severe conditions, such as extreme drought periods, waterbirds relocate to the SLS to seek suitable habitats. In response to habitat conditions altered by variable hydrology, waterbirds adjust their distribution patches according to their habitat needs to adapt to environmental changes, thereby maintaining the diversity of metacommunities within the region. At the patch level, the habitat of the patch determines the community composition of waterbirds, while at the regional level, due to changes in habitat patch conditions, the extinction‐colonization interactive process indicates that waterbirds shift between different habitat patches to obtain habitat and food resources and maintain waterbird diversity in the region.

Because of their high mobility, changing their distribution areas in search of better resources is an important strategy for waterbirds to adapt to changing environments and maintain diversity (Wen et al. 2016). Waterbirds adapt to changing environments by adjusting their behavior and diet to maintain their diversity. For instance, studies have shown that geese change their diet and forage habitat composition to adapt to changes in food and habitat resources (Aharon‐Rotman et al. 2017). Furthermore, shortages of food and habitat caused by hydrological changes may reduce the ability of waterbirds to obtain sufficient energy for adequate winter maintenance and preparation for migration, which may affect winter survival and subsequent reproduction (Aharon‐Rotman et al. 2016; Morrissette et al. 2010; Wang et al. 2013). In future studies, changes in the behavior and energy budgets of waterbirds under a changing environment that is influenced by the hydrological regime should be studied in this region.

### 4.4 Implications for Conservation and Limitations

**FIGURE 8:**
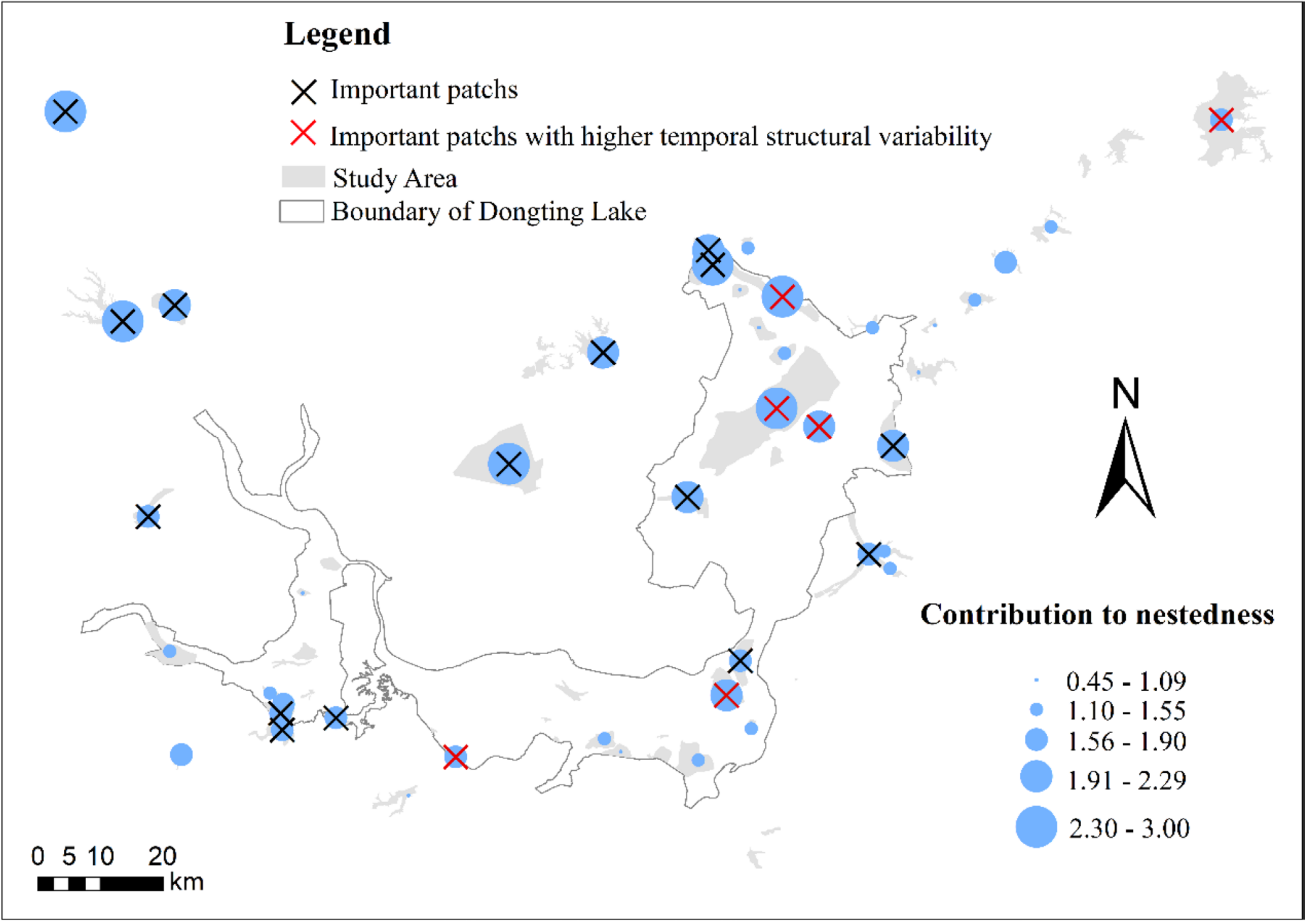
The importance and variability of habitat patches in the study area. The red symbol indicates a higher temporal structural variability of habitat patches.

This study advances the understanding of how hydrological regime influences waterbird community diversity. Under the context of drastic hydrological regime changes, our study provides insights for the conservation and management of waterbird diversity in the middle and lower Yangtze River floodplain. Given the results of the study, the following points should be considered when formulating the management protection strategies for wintering waterbirds. Firstly, it is necessary to enhance habitat management in areas of high importance and temporal structural variability (Figure 8). Secondly, according to the results, water retention is recommended in DTL during the drought year. Adaptive water‐level management in the sub‐lakes is also recommended for DTL; this could create annually stable and favorable habitats for wintering waterbirds (Guan et al. 2016; S. Zhang, Zhang, et al. 2021; Zhu et al. 2022). However, detailed water‐level management plans require further research. Thirdly, our findings emphasize the need for management and protection for SLS, and reducing human disturbance in the framework of enhancing wetland management is encouraged.

This study has certain limitations. Firstly, while our study covers the major distribution areas of waterbirds based on the size and protection status of wetlands, it neglects some habitats such as agricultural areas and rivers. This could lead to an underestimation of the importance of surrounding habitats of DTL for waterbirds, particularly in terms of their overall capacity to support certain functional groups that might utilize these landscapes. Secondly, the four typical hydrological regimes may not fully capture the impacts of long‐term hydrological changes on waterbird diversity, which limits our understanding of the distribution and abundance dynamics of waterbirds under long‐term hydrological changes. Thirdly, although the study includes the major environmental variables, some potential and more detailed factors (e.g., different types of human activity interference index) are not included due to data availability constraints. This may constrain the comprehensive interpretation of the effects of the environment on waterbird diversity.

These findings provide the following insights for future research: (1) Establishing a long‐term waterbird monitoring network to investigate the effects of hydrological regime on waterbird diversity over long‐term time series, while increasing monitoring frequency, habitat types, and spatial coverage; (2) Considering more environmental variables to further reveal the relationship between the environmental variables and waterbird diversity. For instance, the detailed human disturbance factors could be included to differentiate the impacts of various human activities on waterbird diversity; (3) Conducting hydrological control experiments to explore specific parameters for adaptive water‐level management; (4) Satellite tracking data, behavior, energy budgets, and dietary data of waterbirds could be integrated to verify the impact of environmental changes on waterbird distribution patterns under shifting hydrological regimes and investigate differential adaptation strategies among waterbird functional groups to hydrological variability.

## 5 Conclusions

In this study, we investigated the effects of environmental factors on waterbird diversity and underlying processes in Dongting Lake and its surrounding lakes under different hydrological regimes. Our findings suggest that habitat area and human disturbance are important factors that affect waterbird diversity significantly. Hydrological regime primarily influences waterbird diversity indirectly by affecting the composition of habitat, while wetland area determines the total area of habitats. Waterbirds shift their distribution areas in response to habitat changes induced by hydrological regime changes, thereby maintaining their diversity within the region. The subregions with higher variations in habitat composition under the four typical hydrological regimes also exhibited considerable changes in waterbird diversity and community composition. In general, the diversity of waterbirds in EDTL was more sensitive to the hydrological regime, whereas the diversity of SDTL and WDTL remained relatively stable. In the context of changing hydrological conditions, EDTL must be a key area of focus. In addition, future conservation and management should pay more attention to the SLS, which serves as a refuge for waterbirds and plays an important role in maintaining a health network of wetlands across the Dongting Lake area.

## Author Contributions

**Siqi Zhang:** conceptualization (equal), formal analysis (equal), methodology (equal), visualization (lead), writing – original draft (lead), writing – review and editing (equal). **Pingyang Zhang:** conceptualization (equal), investigation (equal), methodology (equal), writing – review and editing (equal). **Yeai Zou:** conceptualization (equal), funding acquisition (equal), supervision (equal), writing – review and editing (equal). **Dongmei Li:** investigation (equal), methodology (equal). **Feng Li:** formal analysis (equal), funding acquisition (equal). **Zhengmiao Deng:** conceptualization (equal), methodology (equal). **Jing Zeng:** investigation (equal), writing – review and editing (equal). **Shengze Wang:** investigation (equal), methodology (equal). **Tao Wu:** investigation (equal), methodology (equal). **Yucheng Song:** data curation (equal), investigation (equal). **Feiyun Li:** data curation (equal), investigation (equal). **Wei Xia:** data curation (equal), investigation (equal). **Yonghong Xie:** funding acquisition (equal), resources (equal), supervision (equal).

## Conflicts of Interest

The authors declare no conflicts of interest.

## Supporting information


**Appendix S1:** ece372396‐sup‐0001‐AppendixS1.doc.

## Data Availability

Relevant data in this study will be available via Dryad: https://doi.org/10.5061/dryad.kh18932n1.
