# Supplementary material for: Waterbird Diversity Patterns Under Varied Hydrological Regimes in Dongting Lake and Surrounding Lakes
Source: Ecol Evol. 2025 Oct 27;15(10):e72396. doi: 10.1002/ece3.72396 (PMC12559672; doi:10.1002/ece3.72396)
Supplement: Supplementary file 1 — Appendix S1: ece372396‐sup‐0001‐AppendixS1.doc. [file ECE3-15-e72396-s001.doc]

**Supplementary Material**

**Supplementary material 1: sub-lakes in Dongting lake and the surrounding lakes**


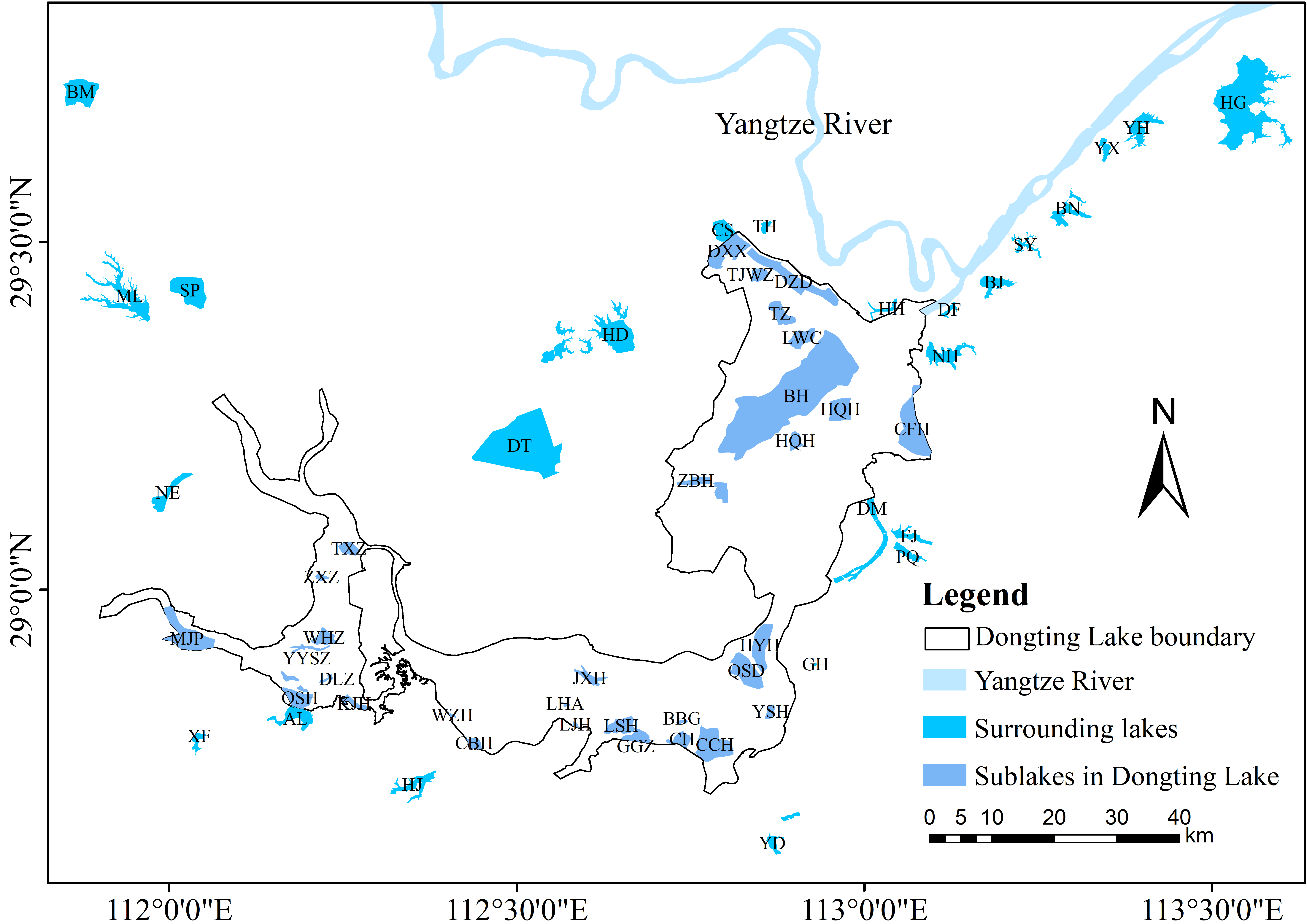


**Fig. S1** Sub-lakes within Dongting lake and the surrounding lakes. The sub-lakes within Dongting Lake are shown in the figure. The locations and names of the surrounding lake also marked on the map.

**Table S1** Sub-lakes in Dongting Lake and the disturbance level.

| **Sites** | **Longitude** | **Latitude** | **Area (km^2^)** | **Disturbance** | **Missing Data** | **Group** |
| --- | --- | --- | --- | --- | --- | --- |
| DXX | 112.8096 | 29.49011 | 14.93 | 1 | / | EDTL |
| DZD | 112.91 | 29.44397 | 21.66 | 2 | / | EDTL |
| TJWZ | 112.8486 | 29.45397 | 4.07 | 3 | / | EDTL |
| TZ | 112.8761 | 29.39938 | 7.64 | 3 | / | EDTL |
| LWC | 112.9127 | 29.36229 | 7.58 | 3 | / | EDTL |
| CFH | 113.0693 | 29.22905 | 24.44 | 3 | / | EDTL |
| BH | 112.9015 | 29.28316 | 145.46 | 1 | / | EDTL |
| ZBH | 112.7729 | 29.15522 | 4.40 | 3 | / | EDTL |
| HQH | 112.9629 | 29.25698 | 10.05 | 2 | / | EDTL |
| HYH | 112.8494 | 28.91925 | 13.48 | 2 | / | SDTL |
| QSD | 112.8293 | 28.86957 | 15.14 | 3 | / | SDTL |
| CCH | 112.7885 | 28.77594 | 19.38 | 2 | / | SDTL |
| GGZ | 112.677 | 28.78799 | 5.23 | 2 | / | SDTL |
| YSH | 112.865 | 28.82134 | 2.18 | 2 | / | SDTL |
| CH | 112.7375 | 28.78582 | 4.58 | 2 | LR/NR | SDTL |
| CBH | 112.4391 | 28.78064 | 2.91 | 2 | / | SDTL |
| LHA | 112.5693 | 28.8363 | 0.53 | 1 | ED | SDTL |
| LSH | 112.6534 | 28.8069 | 8.08 | 3 | / | SDTL |
| WZH | 112.4076 | 28.81932 | 0.44 | 3 | ED | SDTL |
| JXH | 112.6038 | 28.8742 | 4.56 | 1 | ER/LR/NR | SDTL |
| LJH | 112.5841 | 28.80608 | 0.54 | 3 | ER/LR/NR | SDTL |
| BBG | 112.7372 | 28.81055 | 0.64 | 1 | ER/LR/NR | SDTL |
| MJP | 112.0271 | 28.93299 | 20.22 | 3 | / | WDTL |
| DLZ | 112.2308 | 28.87465 | 1.08 | 2 | ED | WDTL |
| BBH | 112.1713 | 28.87276 | 1.75 | 1 | / | WDTL |
| LWH | 112.1912 | 28.85632 | 0.84 | 1 | / | WDTL |
| WHZ | 112.2232 | 28.93222 | 5.62 | 2 | ED | WDTL |
| YYSZ | 112.1933 | 28.91722 | 2.64 | 2 | ED | WDTL |
| ZXZ | 112.2189 | 29.01674 | 1.47 | 3 | / | WDTL |
| KJH | 112.2667 | 28.83688 | 2.78 | 3 | / | WDTL |
| QSH | 112.187 | 28.84321 | 8.11 | 2 | / | WDTL |
| TXZ | 112.2587 | 29.05853 | 4.46 | 2 | LR | WDTL |

**Note:** Dongting Lake (DTL) contains two national nature reserves: East Dongting Lake (EDTL) and West Dongting Lake (WDTL). It also contains two provincial nature reserves located within the South Dongting Lake (SDTL) area: the Hengling Lake Reserve and the South Dongting Lake Reserve.

**Table S2** Complete list of sites surveyed in lake groups surrounding the Dongting Lake.

| **No.** | **Name** | **Code** | **Protection level** | **Assignment PL value** | **Missing data** | **Area (km^2^)** |
| --- | --- | --- | --- | --- | --- | --- |
| 1 | Caisang | CS | NNR | 4 | / | 7.182 |
| 2 | Hao River | HH | NNR | 4 | / | 2.574 |
| 3 | Bajiao | BJ | NONE | 1 | / | 7.877 |
| 4 | Datong | DT | NWP | 4 | / | 79.33 |
| 5 | HRDong Series Lake | HD | NWP | 4 | / | 34.375 |
| 6 | Anle | AL | NNR | 4 | / | 9.779 |
| 7 | Shanpo | SP | NONE | 1 | / | 17.943 |
| 8 | Maoli | ML | NWP/CNR | 4 | / | 22.814 |
| 9 | Nan | NH | NNR | 4 | / | 11.657 |
| 10 | Daming Series Lake | DM | NNR | 4 | / | 12.618 |
| 11 | Huanggai | HG | PNR | 3 | / | 74.989 |
| 12 | Ye | YH | NONE | 1 | LR | 10.62 |
| 13 | Yangxi | YX | NONE | 1 | LR | 3.481 |
| 14 | Dongfeng | DF | NONE | 1 | / | 1.726 |
| 15 | Feijia | FJ | NNR | 4 | / | 5.355 |
| 16 | Pingqiao | PQ | NNR | 4 | / | 4.473 |
| 17 | Beimin | BM | CNR | 2 | / | 16.15 |
| 18 | Xifeng | XF | NWP | 4 | / | 2.843 |
| 19 | Niaoer | NE | NWR | 4 | / | 8.271 |
| 20 | Huangjia | HJ | NWP | 4 | / | 8.781 |
| 21 | Yangsha&Dong | YD | NWP | 4 | LR | 4.953 |
| 22 | Tuan | TH | NNR | 4 | / | 1.827 |
| 23 | Baini | BN | NWP | 4 | / | 8.631 |
| 24 | Donggu | GH | NONE | 1 | ER | 0.139 |
| 25 | Songyang | SY | NONE | 1 | / | 3.843 |

The national nature reserve and national wetland park are assigned to 4; Provincial- level nature reserve is assigned to 3; County-level nature reserve is assigned to 2; Lakes that are not protected areas are assigned to 1.

NNR: National Nature Reserve;

PNR: Provincial-level Nature Reserve;

CNR: County-level Nature Reserve;

NWP: National Wetland Park;

**Supplementary material 2: Summary of wintering waterbird.**

**Table S3** Summary of wintering waterbird surveys in study area (2019/2020-2022/2023)

| **Scientific Name** | **Common Name** | **Functional group** | **Mean** |
| --- | --- | --- | --- |
| *Anas zonorhyncha* | Eastern Spot-billed Duck | Dabbling ducks | 12157.75 |
| *Mareca strepera* | Gadwall | Dabbling ducks | 2300.75 |
| *Mareca penelope* | Eurasian Wigeon | Dabbling ducks | 3460.25 |
| *Tadorna ferruginea* | Ruddy Shelduck | Dabbling ducks | 444 |
| *Histrionicus histrioncus* | Harlequin Duck | Dabbling ducks | 1.25 |
| *Sibirionetta formosa* | Baikal Teal (Ⅱ) | Dabbling ducks | 1 |
| *Mareca falcata* | Falcated Duck (NT) | Dabbling ducks | 51771.25 |
| *Anas crecca* | Grenn-winged Teal | Dabbling ducks | 23332 |
| *Anas platyrhynchos* | Mallard | Dabbling ducks | 4920.5 |
| *Spatula clypeata* | Northern Shoveler | Dabbling ducks | 832 |
| *Tadorna tadorna* | Common Shelduck | Dabbling ducks | 400.5 |
| *Anas acuta* | Northern Pintail | Dabbling ducks | 7042 |
| *Aythya nyroca* | Ferruginous Duck (NT) | Diving ducks | 8.5 |
| *Aythya marila* | Greater Scaup | Diving ducks | 30 |
| *Aythya fuligula* | Tufted Duck | Diving ducks | 4927.5 |
| *Aythya ferina* | Common Pochard (VU) | Diving ducks | 2224.5 |
| *Aythya baeri* | Baer's Pochard (Ⅰ,CR) | Diving ducks | 4.25 |
| *Bucephala clangula* | Common Goldeneye | Diving ducks | 52 |
| *Podiceps cristatus* | Great Crested Grebe | Diving fishers | 1156 |
| *Phalacrocorax carbo* | Great Cormorant | Diving fishers | 6353.5 |
| *Mergus merganser* | Common Merganser | Diving fishers | 42.25 |
| *Tachybaptus ruficollis* | Little Grebe | Diving fishers | 1650 |
| *Hydroprogne caspia* | Caspian Tern | Gulls | 0.75 |
| *Chroicocephalus ridibundus* | Black-headed Gull | Gulls | 10048.75 |
| *Chlidonias hybrida* | Whiskered Tern | Gulls | 1.25 |
| *Larus vegae* | Vega Gull | Gulls | 640.75 |
| *Anser albifrons* | Greater White-fronted Goose (Ⅱ) | Herbivores geese | 701.25 |
| *Anser indicus* | Bar-headed Goose | Herbivores geese | 1 |
| *Anser fabalis* | Bean Goose | Herbivores geese | 28434.75 |
| *Anser cygnoid* | Swan Goose (Ⅱ,VU) | Herbivores geese | 695.5 |
| *Anser anser* | Greylag Goose | Herbivores geese | 2696 |
| *Anser erythropus* | Lesser White-fronted Goose (Ⅱ,VU) | Herbivores geese | 2810.75 |
| *Egretta garzetta* | Little Egret | Large wading birds | 795 |
| *Platalea leucorodia* | Eurasian Spoonbill (Ⅱ) | Large wading birds | 5653.25 |
| *Ardea cinerea* | Grey Heron | Large wading birds | 3239.25 |
| *Ardea purpurea* | Purple Heron | Large wading birds | 2.25 |
| *Ardeola bacchus* | Chinese Pond Heron | Large wading birds | 8 |
| *Ardea alba* | Great Egret | Large wading birds | 783.75 |
| *Ciconia boyciana* | Oriental Stork (Ⅰ,EN) | Large wading birds | 2.25 |
| *Ciconia nigra* | Black Stork (Ⅰ) | Large wading birds | 66 |
| *Bubulcus ibis* | Cattle Egret | Large wading birds | 16.5 |
| *Nycticorax nycticorax* | Black-crowned Night Heron | Large wading birds | 354.75 |
| *Ardea intermedia* | Intermediate Egret | Large wading birds | 89.25 |
| *Numenius arquata* | Eurasian Curlew (Ⅱ, NT) | Small wading birds | 11.25 |
| *Tringa ochropus* | Green Sandpiper | Small wading birds | 11.5 |
| *Recurvirostra avosetta* | Pied Avocet | Small wading birds | 4075 |
| *Vanellus vanellus* | Northern Lapwing (NT) | Small wading birds | 4643.75 |
| *Tringa erythropus* | Spotted Redshank | Small wading birds | 5906 |
| *Himantopus himantopus* | Black-winged Stilt | Small wading birds | 74.75 |
| *Calidris alpina* | Dunlin | Small wading birds | 7463.25 |
| *Limosa limosa* | Black-tailed Godwit (NT) | Small wading birds | 80.5 |
| *Charadrius alexandrinus* | Kentish Plover | Small wading birds | 272.25 |
| *Pluvialis squatarola* | Grey Plover | Small wading birds | 6.75 |
| *Vanellus cinereus* | Grey-headed Lapwing | Small wading birds | 6.25 |
| *Actitis hypoleucos* | Common Sandpiper | Small wading birds | 16 |
| *Pluvialis fulva* | Pacific Golden Plover | Small wading birds | 1 |
| *Charadrius dubius* | Little Ringed Plover | Small wading birds | 4.5 |
| *Calidris temminckii* | Temminck's stint | Small wading birds | 2 |
| *Tringa nebularia* | Common Greenshank | Small wading birds | 155.25 |
| *Gallinago gallinago* | Common Snipe | Small wading birds | 19.25 |
| *Grus leucogeranus* | Siberian Crane (Ⅰ,CR) | Tuber feeding birds | 31.75 |
| *Grus monacha* | Hooded Crane (Ⅰ,VU) | Tuber feeding birds | 1 |
| *Grus vipio* | White-naped Crane (Ⅰ,VU) | Tuber feeding birds | 1.25 |
| *Grus grus* | Common Crane (Ⅱ) | Tuber feeding birds | 617.75 |
| *Cygnus columbianus* | Tundra Swan (Ⅱ,LC) | Tuber feeding birds | 7906 |
| *Fulica atra* | Common Coot | Vegetation gleaners | 7511.5 |
| *Gallinula chloropus* | Common Moorhen | Vegetation gleaners | 267.25 |
| *Rallus indicus* | Brown-cheeked Rail | Vegetation gleaners | 1.25 |

**Note:** The common scientific names are based on Zheng (2015). Letters in parentheses indicate threatened waterbirds listed in the IUCN Red List (www.iucnredlist.org): CR, Critically Endangered; EN, Endangered; VU, Vulnerable; NT, Near Threatened. Ⅰ and Ⅱ indicate threatened waterbirds listed in national levels Ⅰ and Ⅱ protected animals in China.

**Supplementary material 3: Daily water level during different hydrological period.**

The daily water level from 2019-2022 showed that generally, the water level decreased gradually from September to December, while the water level decreased from July in 2022. The annual recession pattern of lake was obtained based on its average daily water level in October which is the key month before waterbird arrival (Zhang et al., 2023). Kruskal-Wallis test was used because normality tests held false. Then, the daily water level in October among the study years and the 30-year mean before the operation of TGD was compared. Results showed that the mean water levels in October were significantly different among the four study years and the 30-year mean before the TGD (p < 0.001). Specifically, the mean water level in October 2021 was not significantly different from that of mean before TGD (p > 0.05), while those of 2019 (p < 0.01) and 2022 (p < 0.001) were significantly lower and 2020 (p < 0.01) were significantly higher. In 2022, an extreme drought event occurred throughout the Yangtze River Basin, severely impacting Dongting Lake (Zhang et al., 2024). Therefore, we classify the four years into four different water recession patterns. Specifically, 2019 represent early water recession year (2019/2020 wintering period), 2020 represent late recession year (2020/2021 wintering period), 2021 represent normal recession year (2021/2022 wintering period), 2022 represent extreme drought (2022/2023 wintering period).


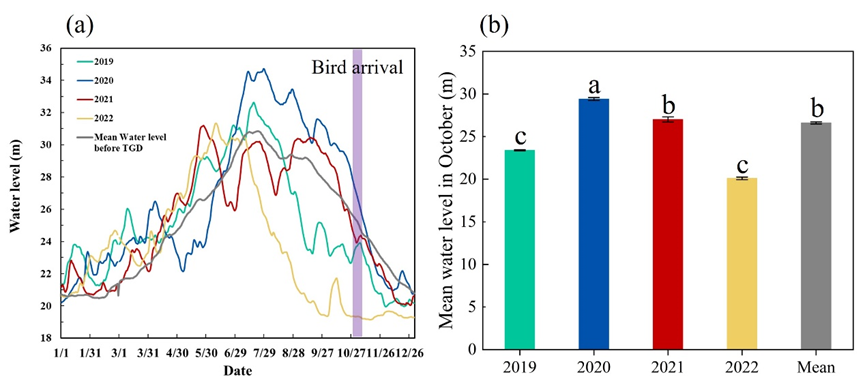


**Fig.S2.** (a) Daily water level in 2019-2022. The grey line indicates the mean water level over 30 years before the operation of TGD (1977-2006). (b) Comparations of mean water levels in October.

**References:**

Zhang, P., Zhang, S., Zou, Y., Wu, T., Li, F., Deng, Z., Zhang, H., Song, Y., Xie, Y., 2023. Integrating suitable habitat dynamics under typical hydrological regimes as guides for the conservation and restoration of different waterbird groups. J. Environ. Manage. 345, 118451. https://doi.org/10.1016/j.jenvman.2023.118451

Zhang, P., Zou, Y., Tao, K., Zhang, S., Li, Feng, Deng, Z., Zeng, J., Xie, Y., Liu, X., Li, Feiyun, 2024. Extreme drought alters waterfowl distribution patterns and spatial niches in floodplain wetlands. Glob. Ecol. Conserv. 51, e02901. https://doi.org/10.1016/j.gecco.2024.e02901

**Supplementary material 4: Environmental variables.**

**Table S4** The description of environmental variables.

| **Classification** | **Environmental variables** | **Description** | **Designated research region** |
| --- | --- | --- | --- |
|  | Area | The total area of each site or lake. | Both (DTL/SLS) |
| Habitat condition | Water area | Water surface area. | Both (DTL/SLS) |
|  | Mudflat area | Mudflat area. | Both (DTL/SLS) |
|  | Vegetation area | The vegetation area in Dongting Lake was the area of *Carex* which could provide the food for herbivores and some tuber feedings, and the area of *Miscanthus* was excluded. As for the relatively small vegetation area, the specific vegetation categories are not distinguished in SLS. | Both (DTL/SLS) |
|  | NDVI | The average NDVI of Vegetation area. Reflect the growth status of vegetation. | Dongting Lake (DTL) |
| Local hydrological condition | Average inundation duration (InDave) | Regions with a larger proportion of water area experienced longer average inundation durations. Additionally, within the same region, variations in the hydrological regime between years also influence the average inundation duration with later recession times correlating to extended durations. | Dongting Lake (DTL) |
|  | Inundation duration < 20 d (InD1) | During the wintering period, the inundation duration less than 20 days are usually non-flooded areas (especially in the surrounding lakes of Dongting Lakes) or areas with higher elevation which are exposed earlier in the recession period. These areas may contain vegetation (it could be *Miscanthus* which distributed in relatively high elevation in Dongting Lake) or mudflat habitats after exposed (Fig. S3). | Both (DTL/SLS) |
|  | Inundation duration 20-70 d (InD2) | During the overwintering period, these areas are gradually exposed with the decline of water levels, and these areas are basically fully exposed by mid-November. These periodically flooded areas form vegetation and mudflat zones after exposure, providing habitats and food sources for a variety of waterbird species (Fig. S3). Based on the stable hydrology conditions of the SLS and the large water surface area, InD1 and InD2 also represent the periodically flooded areas of these lakes, which provided habitats for waterbirds relay on mudflats and vegetation after the water recession. | Both (DTL/SLS) |
| Morphological Characteristics | Shape Index (SI) | The Shape Index is used to reflect the complexity of the lake shape. Larger value reflects more complex shape of the wetland. 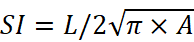. *L* stands for wetland perimeter and A represent the wetland area. | Surrounding lakes (SLS) |
| Mean Water Depth | Mean Water Depth (MWD) | Mean water depth. | Surrounding lakes (SLS) |
| Human disturbance | Disturbance | Ordinal data according to the human disturbance (e.g. road distance, human activities). Detailed information is in the Table S1. | Dongting Lake (DTL) |
|  | Road density (ROD) | Reflect the human disturbance. | Surrounding lakes (SLS) |
|  | Resident density (RED) | Reflect the human disturbance. | Surrounding lakes (SLS) |
| Protection | Protection level | Assign values according to protected area level. Detailed information is in the Table S2. | Surrounding lakes (SLS) |

**Note:** Dongting Lake (DTL) contains two national nature reserves: East Dongting Lake (EDTL) and West Dongting Lake (WDTL). It also contains two provincial nature reserves located within the South Dongting Lake (SDTL) area: the Hengling Lake Reserve and the South Dongting Lake Reserve. Most of the areas within Dongting Lake fall within the scope of these nature reserves. Therefore, the protection level has not been taken into consideration when analyzing the diversity factors within Dongting Lake.


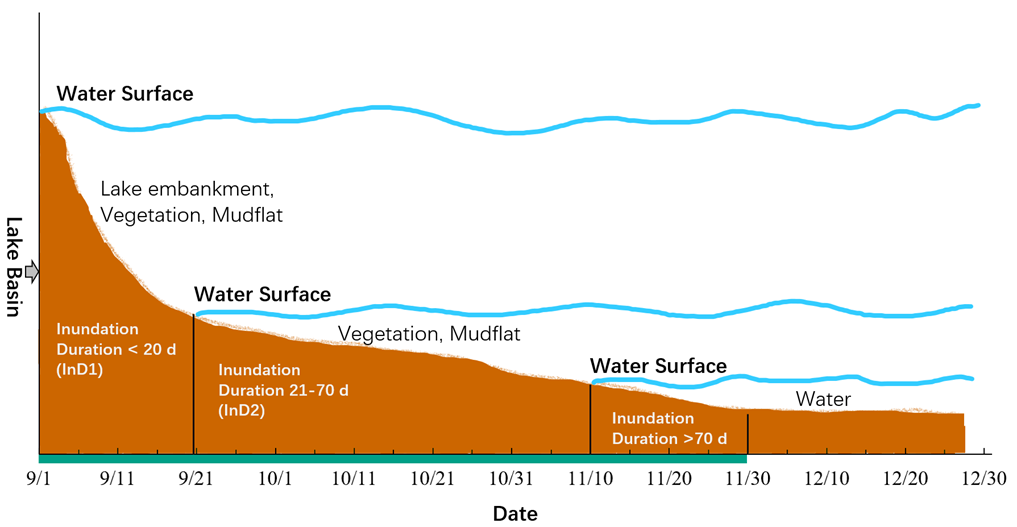


**Fig. S3** Local hydrology index calculation

**Table S5**. Variables in Dongting Lake

| No. | Variables | VIF (with all variables) | VIF (after removing) |
| --- | --- | --- | --- |
| 1 | Area | 21.58 | Removed |
| 2 | Water area | 5.90 | 2.06 |
| 3 | Mudflat area | 5.49 | 4.92 |
| 4 | Vegetation area | 5.96 | 4.79 |
| 5 | InD 1 | 4.16 | 3.13 |
| 6 | InD 2 | 6.91 | 5.34 |
| 7 | InDave | 2.33 | 2.05 |
| 8 | Disturbance | 1.17 | 1.17 |
| 9 | NDVI | 1.87 | 1.87 |

**Table S6. Variables in Surrounding Lakes**

| No. | Variables | VIF (with all variables) | VIF (after removing) |
| --- | --- | --- | --- |
| 1 | Area | 105.87 | Removed |
| 2 | Water area | 74.53 | 4.43 |
| 3 | Mudflat area | 3.58 | 2.37 |
| 4 | Vegetation area | 3.98 | 3.43 |
| 5 | InD 1 | 1.91 | 1.59 |
| 6 | InD 2 | 7.63 | 7.58 |
| 7 | ROD | 2.74 | 2.12 |
| 8 | RED | 2.01 | 1.77 |
| 9 | Protection | 1.48 | 1.40 |
| 10 | SI | 1.49 | 1.42 |
| 11 | MWD | 1.72 | 1.66 |

**Supplementary material 5: components of temporal dissimilarity (beta diversity) of species-patch networks representing metacommunities**

**Table S7** the components of temporal dissimilarity (beta diversity) of species-patch networks representing metacommunities

| Measure | Definition | Ecological process |
| --- | --- | --- |
| β_Temporal_ | Dissimilarity of species-patch links between t1 and t2 | Changes in species distribution pattern at regional scale between t1 and t2 |
| β_Local_ | Dissimilarity of species-patch links introduced by link composition changes in shared parts (species and patch) between t1 and t2 | Colonization-extinction processes of species at local patches. These species and patches neither contributed to the changes in the regional species pool nor patch composition between the t1 and t2 |
| β_Regional_ | Dissimilarity of species-patch links introduced by changes of regional species pool only | Colonization-extinction processes of species at a regional scale. New species colonized into the patches which existed in t1 and/or the species that established in t1 but regional extinct in t2 |
| β_Landscape_ | Dissimilarity of species-patch links which only driven by changes of patch compositions | Natural or human-mediated gains or losses of patches. Old species in t1 colonized into the gained patches and/or old species (but these species still exist in t2) extinct from the lost patches. |
| β_RL_ | Dissimilarity of species-patch links introduced by changes of patches and regional species pool simultaneously | Species gains/losses process and patch gains/loss process cooccurred. Some patches lost in t2, the unique species in these lost patches extinct regionally in t2; and some patches gained in t2, the new unique species from out of region colonized into these gained patches in t2 |
| β_Extinction_ | Dissimilarity of species-patch links due to the loss of soecies-patch links | Including all local and regional extinction events across time |
| β_Colonization_ | Dissimilarity of species-patch links due to the gain of species-patch links | Including all local and regional colonization events across time |

Note: the table is cited from Li et al. (2023).

**References:**

Li, H., Holyoak, M., Xiao, Z., 2023. Disentangling spatiotemporal dynamics in metacommunities through a species‐patch network approach. Ecol. Lett. 26, 1261–1276. https://doi.org/10.1111/ele.14243

**Supplementary material 6: Average diversity index values of sub-regions during four hydrological regimes**

**Table S8** Average abundance of sub-regions during four hydrological regimes

| Abundance | EDTL | SDTL | WDTL | SLS |
| --- | --- | --- | --- | --- |
| Extremely drought | 5295.7±1482.6aA | 2133.9±528.1aA | 3415.0±1831.9aA | 6712.9±2474.1aA |
| Early recession | 13627.8±5336.4aA | 2175.8±855.3aAB | 2230.±963.0aAB | 3220.5±1600.8abB |
| Normal recession | 14520.9±4905.9aA | 2323.0±742.0aAB | 1739.8±1063.7aA | 3035.6±1165.7abB |
| Late recession | 10212.1±3923.6aA | 1278.4±568.7aAB | 1490.1±825.6aAB | 540.6±189.6bB |

**Table S9** Average richness of sub-regions during four hydrological regimes

| Richness | EDTL | SDTL | WDTL | SLS |
| --- | --- | --- | --- | --- |
| Extremely drought | 17.6±3.1aA | 9.8±0.8aA | 10.7±3.2aA | 13.5±0.8aA |
| Early recession | 17.6±2.9aA | 9.7±1.6aA | 9.6±2.0aA | 10.1±1.5abA |
| Normal recession | 15.3±3.2aA | 15.0±2.1aA | 10.2±2.1aA | 9.5±1.4abA |
| Late recession | 17.8±3.4aA | 9.1±2.0aAB | 9.2±1.3aAB | 6.7±0.9bB |

**Table S10** Average SHDI of sub-regions during four hydrological regimes

| SHDI | EDTL | SDTL | WDTL | SLS |
| --- | --- | --- | --- | --- |
| Extremely drought | 1.44±0.22aA | 1.46±0.10aA | 1.17±0.16aA | 1.34±0.12aA |
| Early recession | 1.37±0.26aA | 1.37±0.18aA | 1.08±0.21aA | 1.12±0.12aA |
| Normal recession | 1.22±0.25aA | 1.76±0.12aA | 1.20±0.13aA | 1.17±0.13aA |
| Late recession | 1.76±0.16aA | 1.28±0.19aAB | 1.19±0.20aAB | 1.07±0.12aB |

**Table S11** Average pielou index of sub-regions during four hydrological regimes

| Pielou | EDTL | SDTL | WDTL | SLS |
| --- | --- | --- | --- | --- |
| Extremely drought | 0.55±0.05aA | 0.66±0.04aA | 0.65±0.06aA | 0.59±0.03aA |
| Early recession | 0.48±0.07aA | 0.65±0.09aA | 0.53±0.08aA | 0.57±0.04aA |
| Normal recession | 0.51±0.07aA | 0.68±0.05aA | 0.58±0.05aA | 0.61±0.04aA |
| Late recession | 0.66±0.04aA | 0.68±0.07aA | 0.58±0.09aA | 0.65±0.04aA |

**Note:** Mean value ± SE. Uppercase letters indicate comparisons of data within the same row, while lowercase letters indicate comparisons of data within the same column.


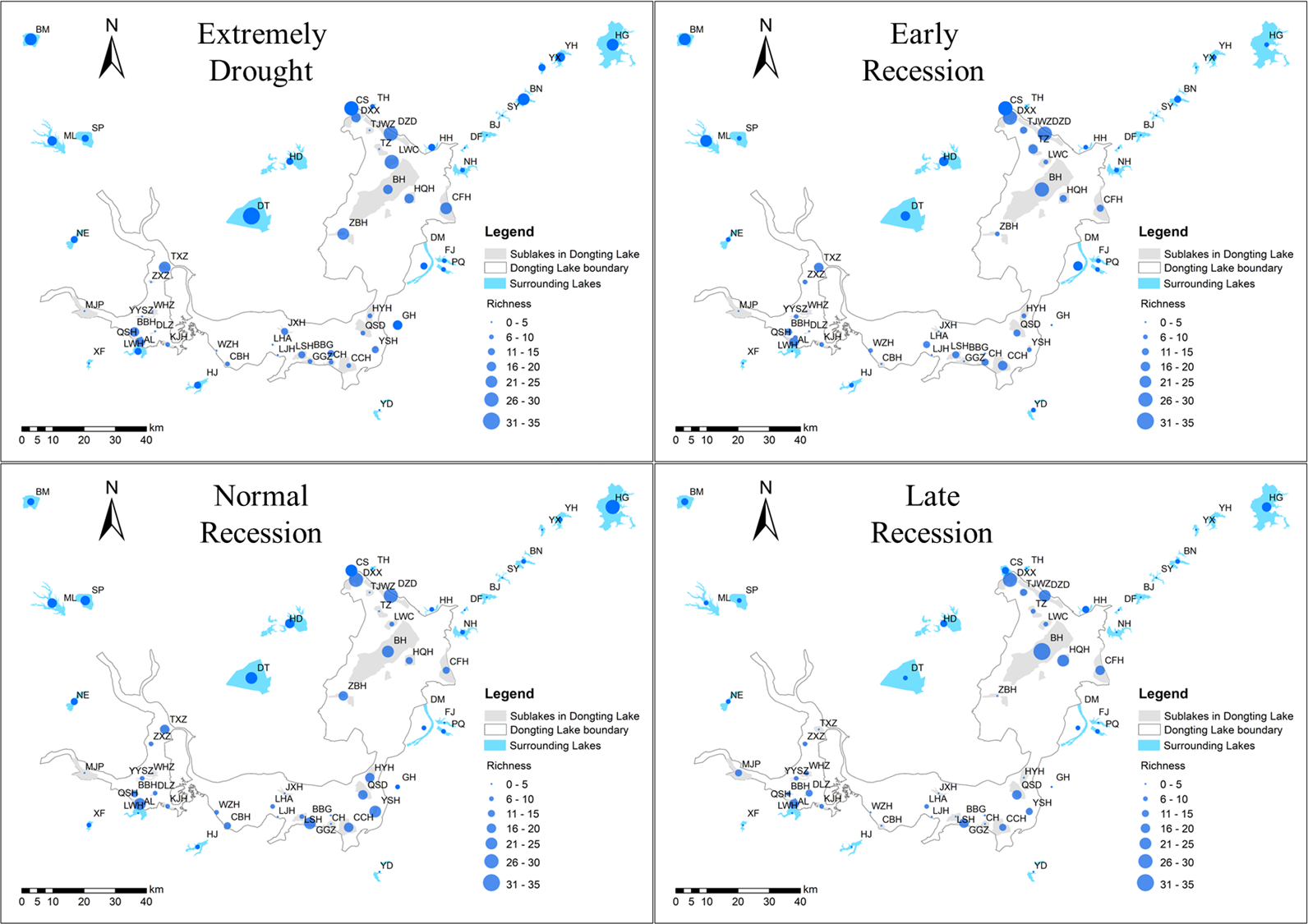


**Fig. S4** Richness values in study area.


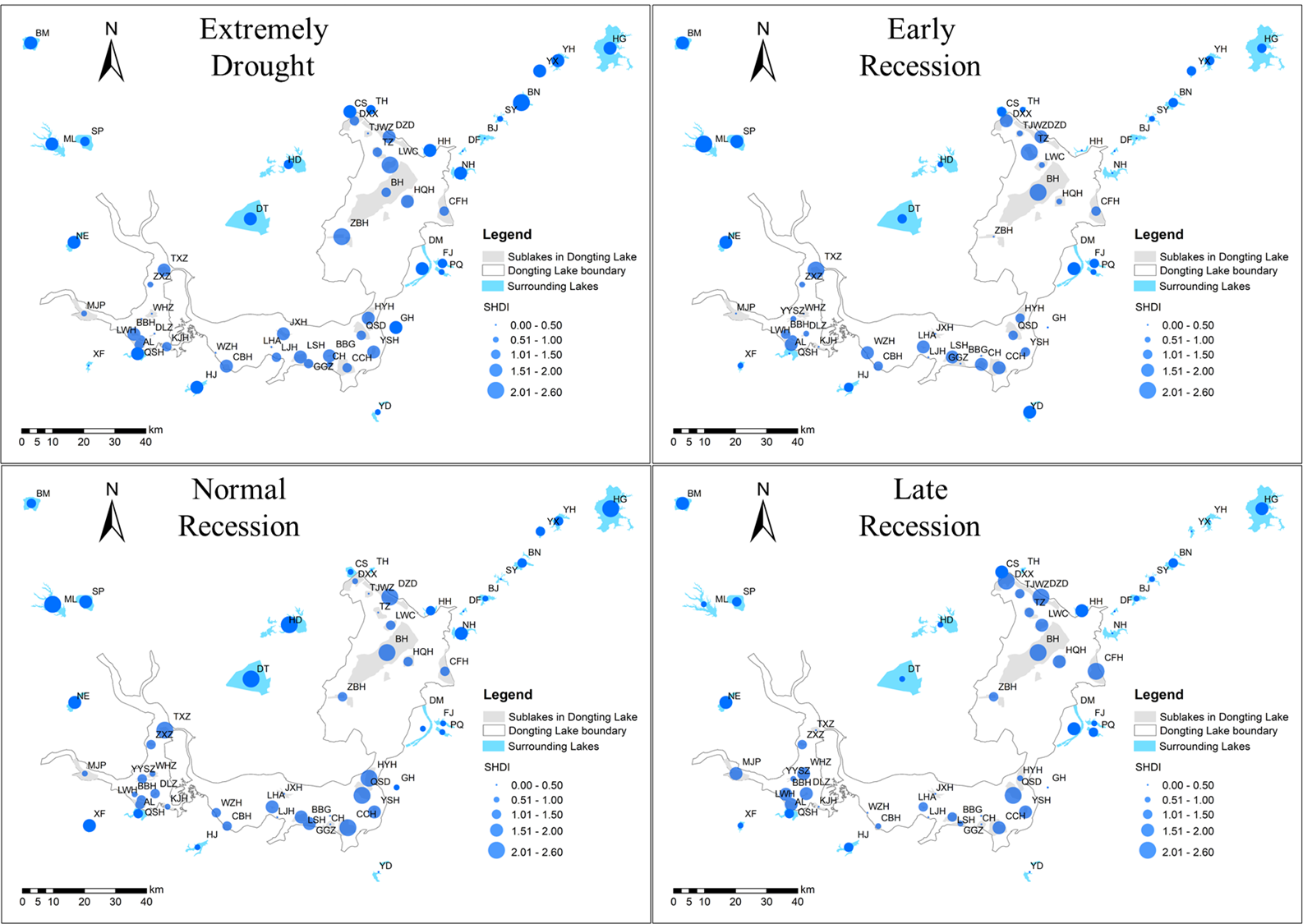


**Fig. S5** SHDI values in study area.


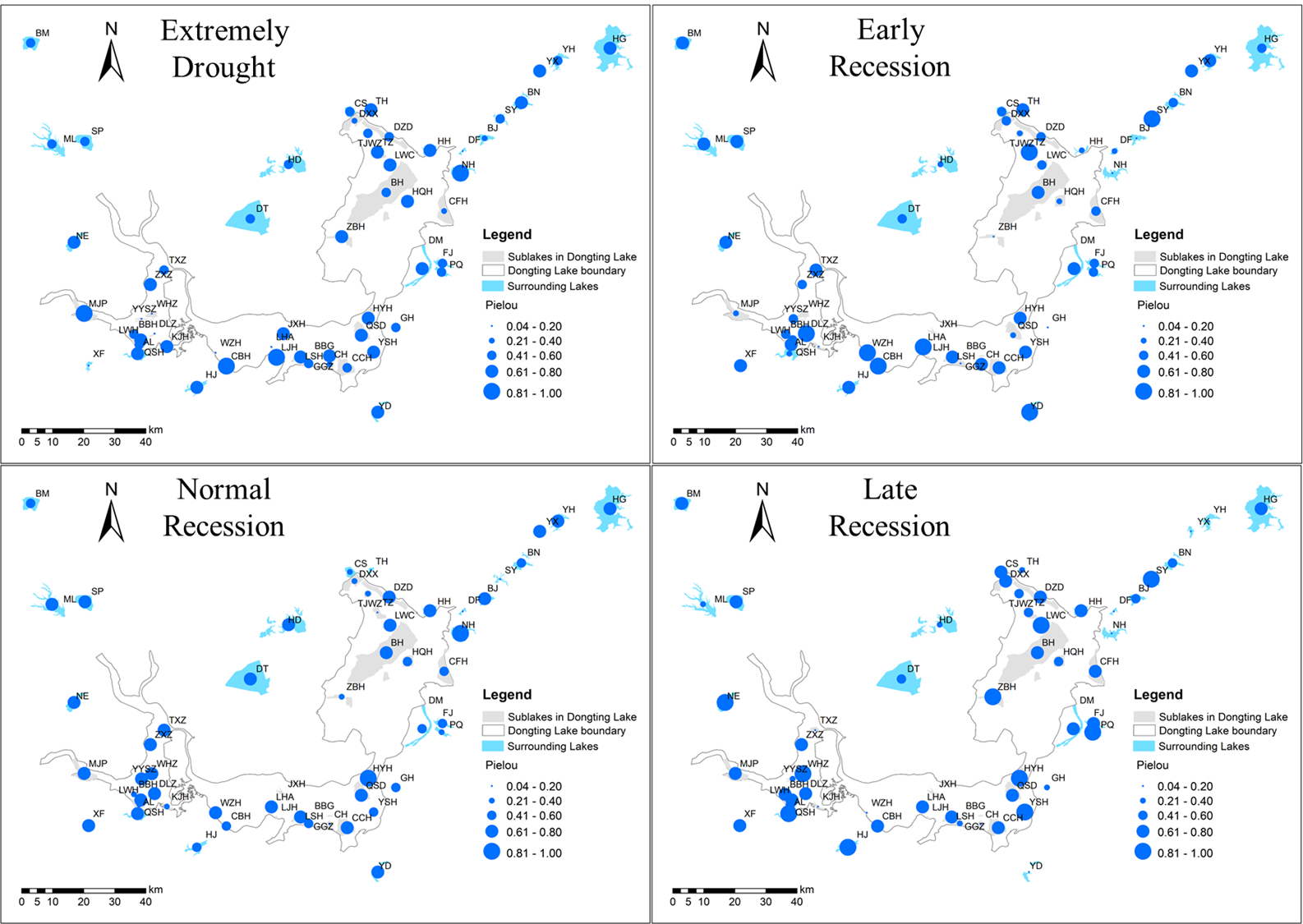


**Fig. S6** Pielou index values in study area.


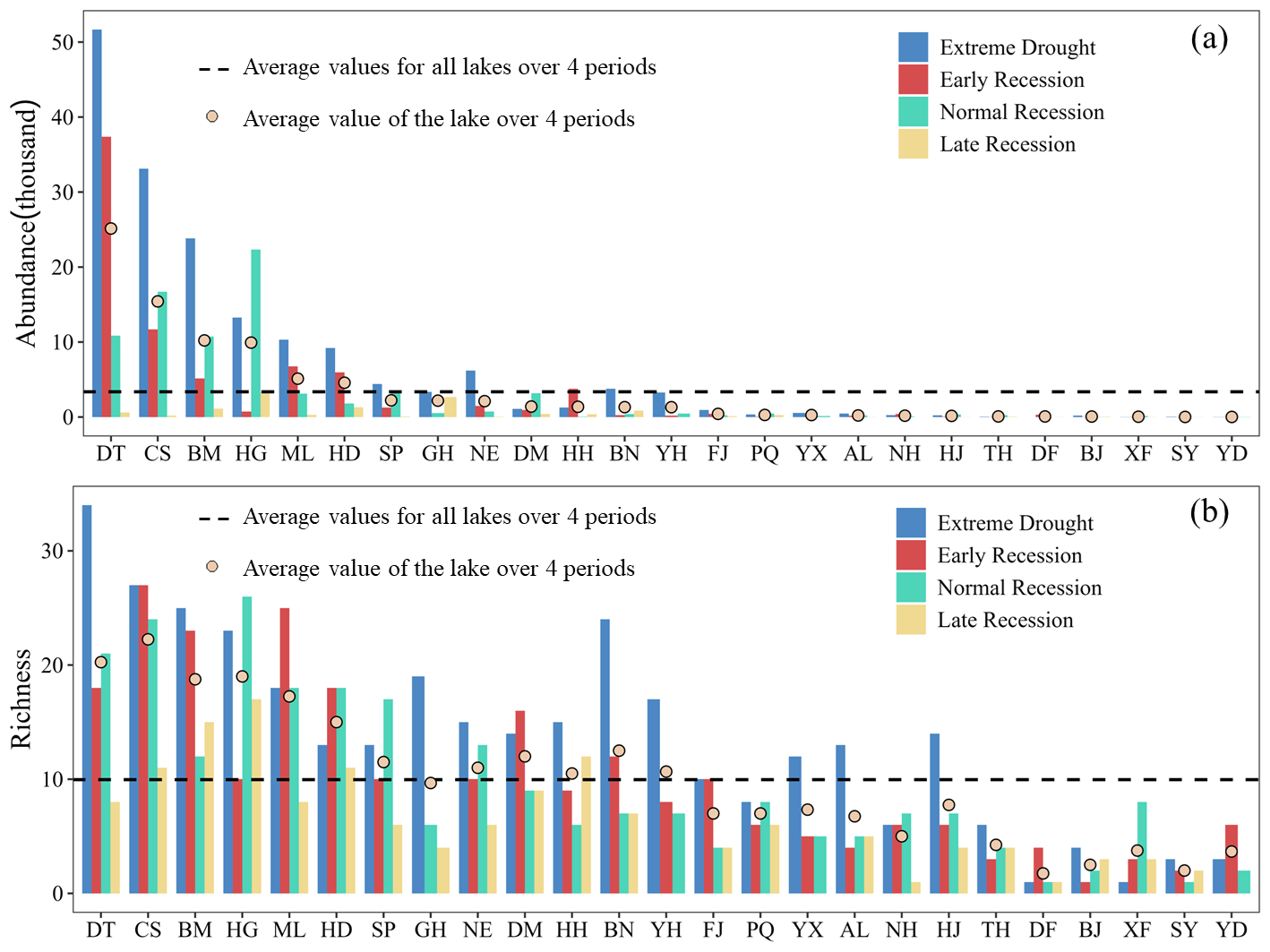


**Fig. S7** Abundance and richness distribution of waterbirds in surrounding lakes (SLS).


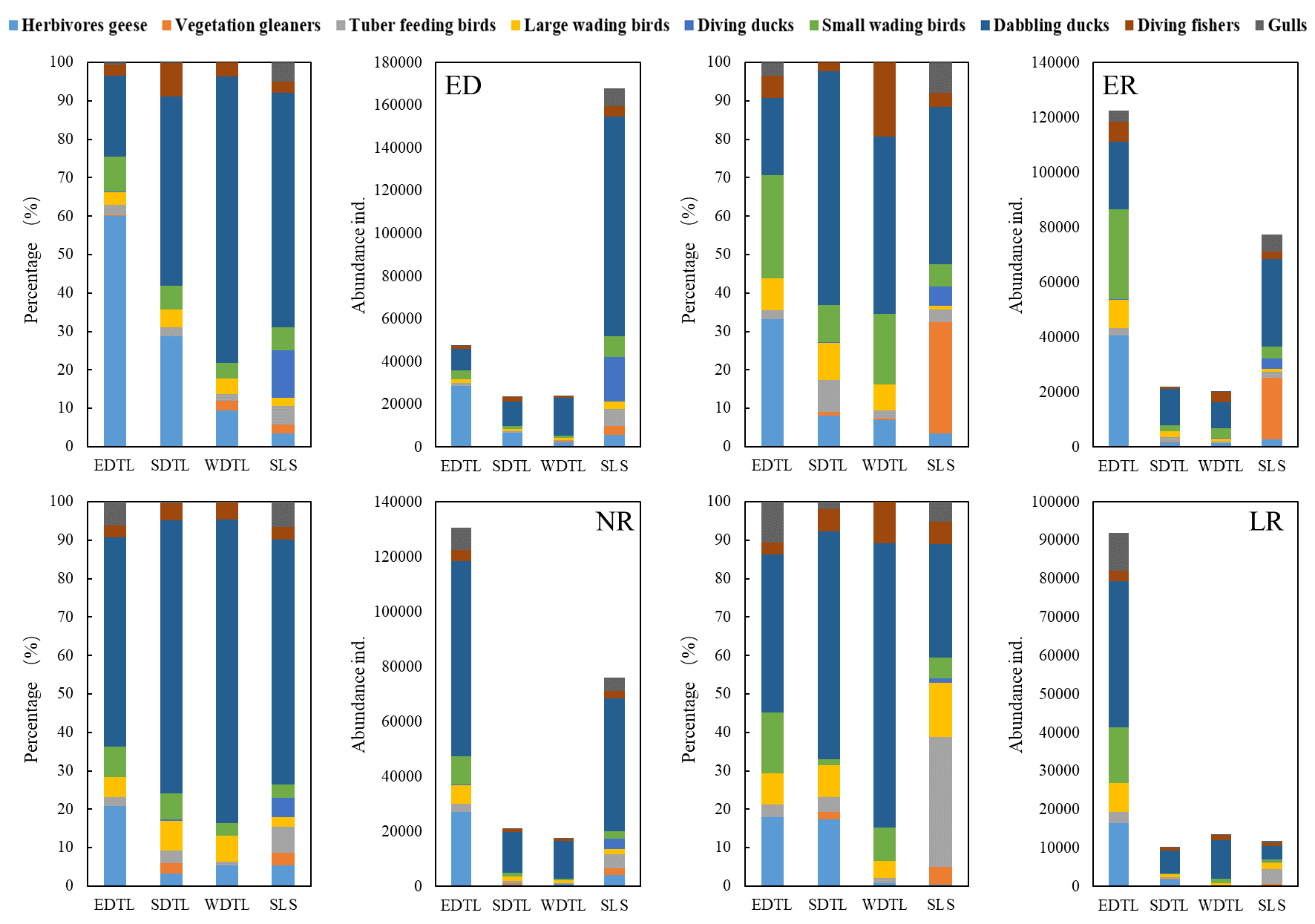


**Fig. S8** Functional groups composition during different hydrological regime

**Supplementary material 7: Community composition in sub-regions during different hydrological period at the local scale.**


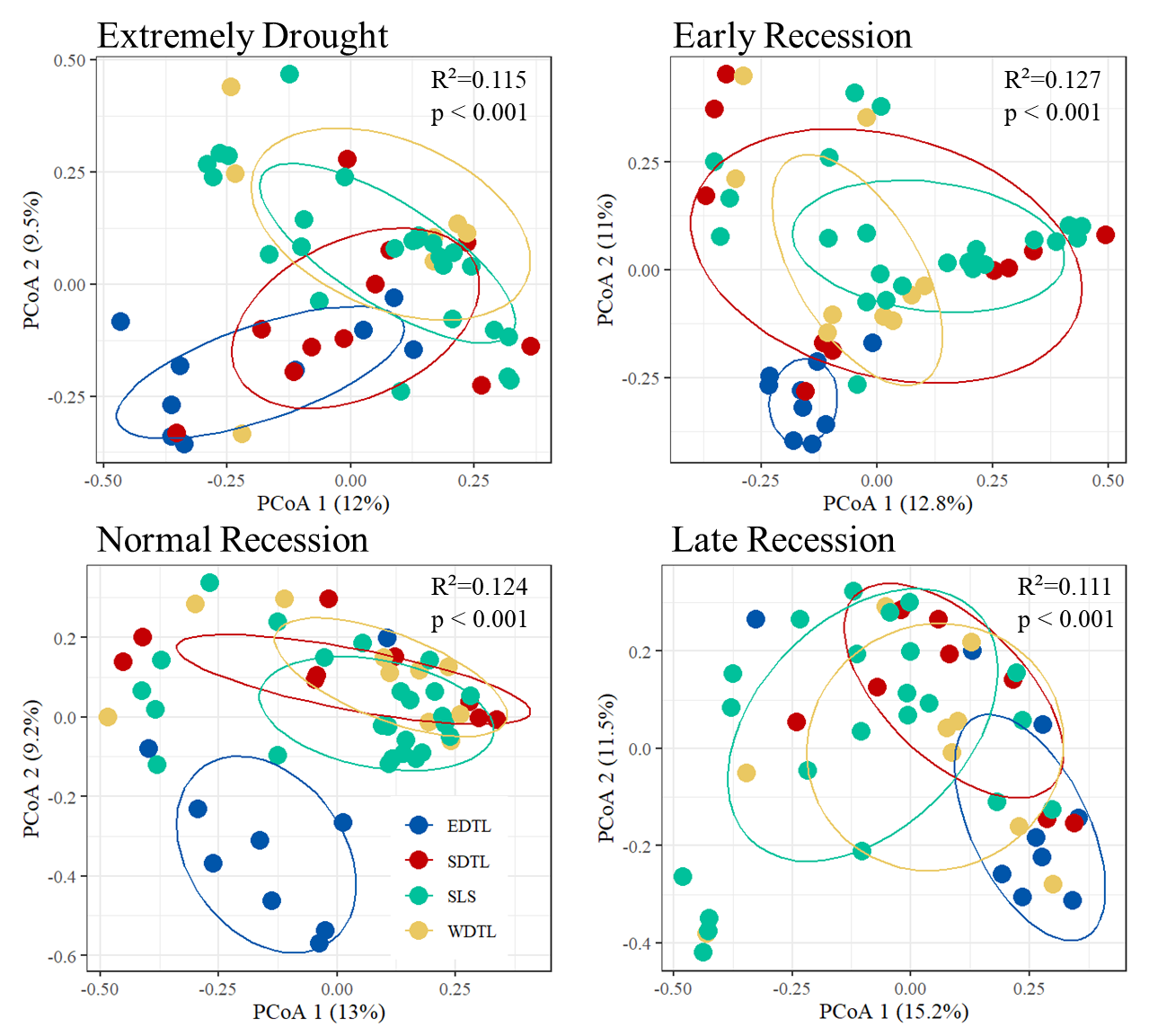


**Fig. S9** PCoA ordination map based on bray-cruits distance of species abundance with the result of the PERMANOVA test between different sub-region at local scale.

**Table S12 Pairwise test of PERMANOVA results between different sub-regions**

| Hydrological regime |  | EDTL  v.s  SDTL | EDTL  v.s  WDTL | EDTL  v.s  SLS | SDTL  v.s  WDTL | SDTL  v.s  SLS | WDTL  v.s  SLS |
| --- | --- | --- | --- | --- | --- | --- | --- |
| Extremely Drought | R² | 0.124 | 0.153 | 0.101 | 0.067 | 0.041 | 0.043 |
|  | F | 2.549 | 2.530 | 3.603 | 1.145 | 1.464 | 1.339 |
|  | *p* | **0.009** | **0.019** | **0.001** | 0.286 | 0.070 | 0.113 |
| Early Recession | R² | 0.162 | 0.164 | 1.12 | 0.056 | 0.038 | 0.048 |
|  | F | 3.294 | 3.139 | 4.245 | 1.006 | 1.299 | 1.548 |
|  | *p* | **0.001** | **<0.001** | **<0.001** | 0.412 | 0.190 | 0.067 |
| Normal Recession | R² | 0.164 | 0.164 | 0.096 | 0.05 | 0.047 | 0.054 |
|  | F | 3.148 | 3.326 | 3.382 | 0.903 | 1.579 | 1.878 |
|  | *p* | **0.001** | **0.001** | **0.001** | 0.545 | **0.036** | **0.006** |
| Late Recession | R² | 0.101 | 0.115 | 0.085 | 0.055 | 0.052 | 0.057 |
|  | F | 1.684 | 2.08 | 2.683 | 0.867 | 1.541 | 1.755 |
|  | *p* | 0.061 | **0.02** | **0.01** | 0.609 | 0.072 | 0.054 |


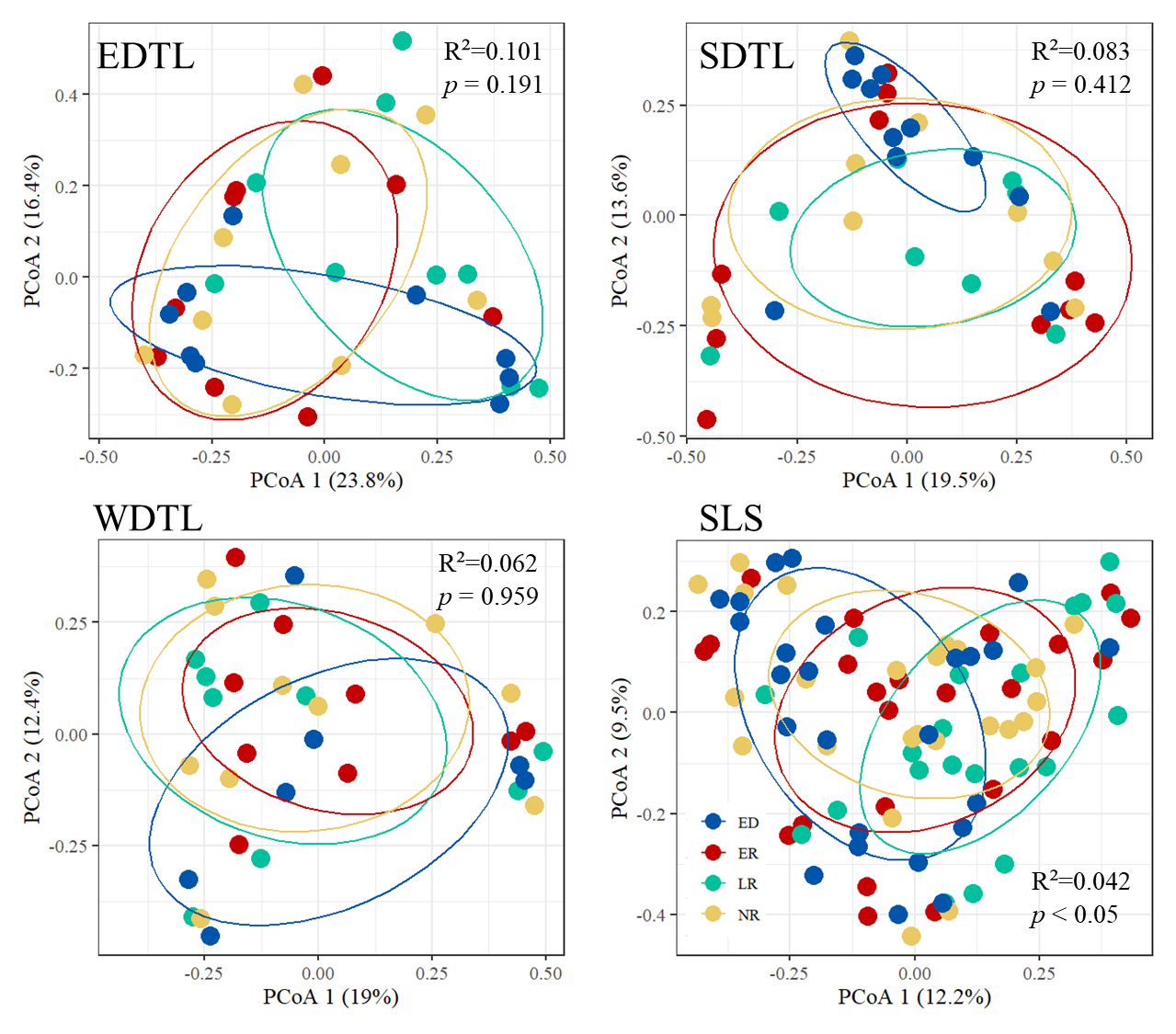


**Fig. S10** PCoA ordination map based on bray-cruits distance of species abundance with the result of the PERMANOVA test between different hydrological regime.

**Table S13 Pairwise test of PERMANOVA results between different hydrology regime.**

| Groups |  | Extremely Drought  v.s  Early Recession | Extremely Drought  v.s  Normal Recession | Extremely Drought  v.s  Late Recession | Early Recession  v.s  Normal Recession | Early Recession  v.s  Late Recession | Normal Recession  v.s  Late Recession |
| --- | --- | --- | --- | --- | --- | --- | --- |
| EDTL | R² | 0.059 | 0.066 | 0.080 | 0.046 | 0.082 | 0.082 |
|  | F | 0.995 | 1.122 | 1.387 | 0.776 | 1.433 | 1.437 |
|  | *p* | 0.475 | 0.475 | 0.323 | 0.672 | 0.323 | 0.323 |
| SDTL | R² | 0.06 | 0.061 | 0.072 | 0.042 | 0.048 | 0.054 |
|  | F | 1.223 | 1.162 | 1.311 | 0.745 | 0.807 | 0.848 |
|  | *p* | 0.514 | 0.514 | 0.514 | 0.672 | 0.672 | 0.672 |
| WDTL | R² | 0.052 | 0.051 | 0.063 | 0.04 | 0.028 | 0.028 |
|  | F | 0.761 | 0.806 | 0.936 | 0.706 | 0.458 | 0.498 |
|  | *p* | 0.972 | 0.972 | 0.972 | 0.972 | 0.972 | 0.972 |
| SLS | R² | 0.023 | 0.013 | 0.046 | 0.015 | 0.036 | 0.042 |
|  | F | 1.083 | 0.645 | 2.164 | 0.693 | 1.649 | 1.978 |
|  | *p* | 0.495 | 0.935 | **0.011** | 0.935 | 0.065 | **0.011** |

**Supplementary material 8: Variation of habitat area under different hydrological regimes**


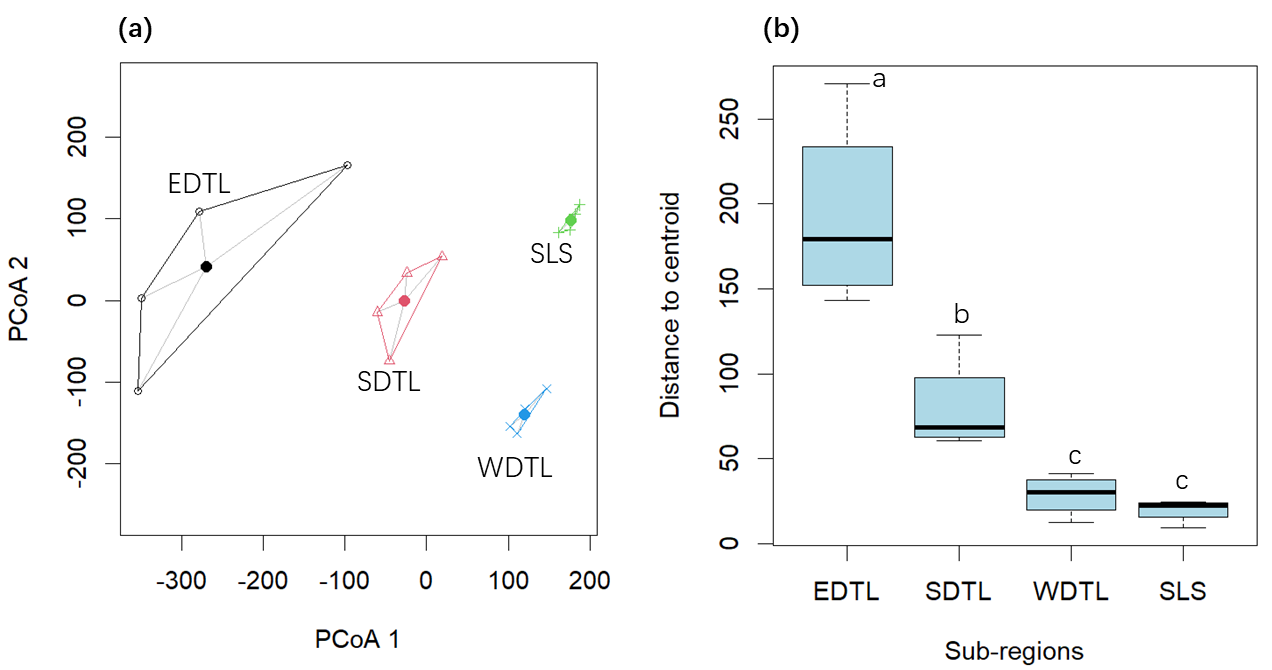


**Fig. S11** Habitat composition variation during different hydrological regime. (a) PCoA ordination map based on euclidean distance of habitat area; (b) distance to group centroid between sub-regions. All tests were performed with 999 permutations.


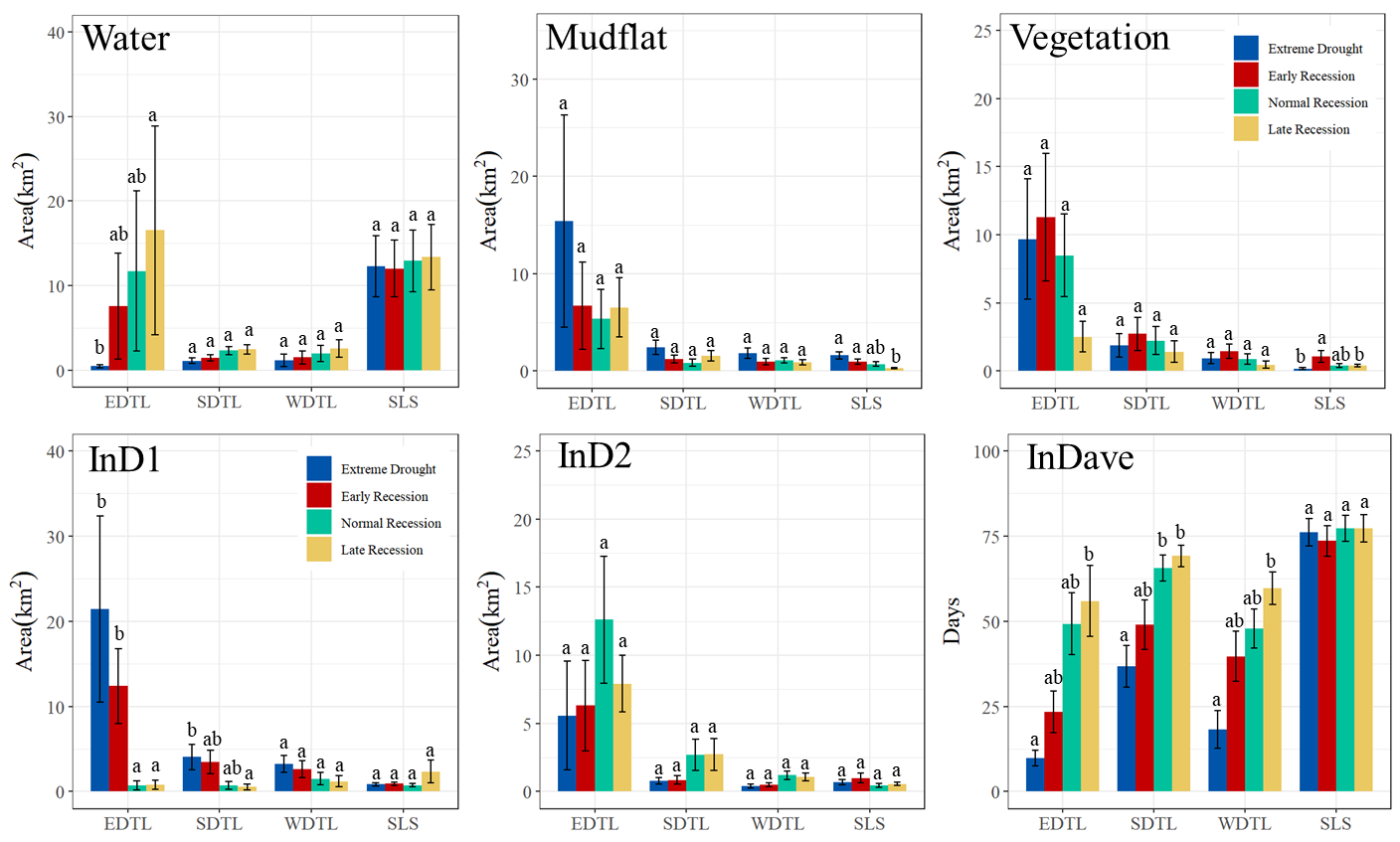


**Fig. S12** Variations of habitat area and local hydrology indices under different hydrological regimes

**Supplementary material 9: the important site of waterbird-habitat patch network**

The importance of each node within the waterbird-habitat patch network are assessed by the index of *contribution to nestedness (cnodf)*. Habitat patches with the top 21 (50%) highest *cnodf* values are identified as important habitat patches in the network. Moreover, patches with the number of species not exceeding 10% of the total recorded species for that year are excluded from the list of important patches and are replaced by the next ones in line.

To evaluate the important and stable sites versus the important and unstable sites: calculate the average values across four periods and compute the coefficient of variation (CV) for each node to measure its variability. Patches with CVs ranking among the top 21 (50%) are considered patches with higher temporal structural variability.


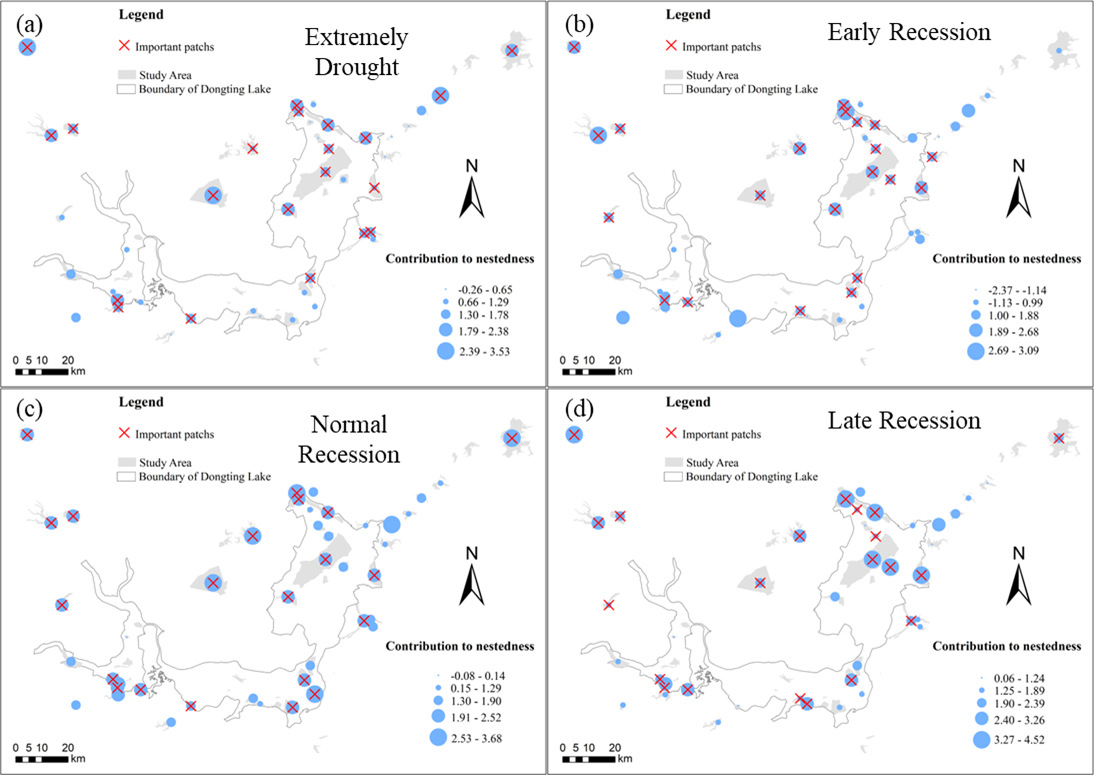


**Fig. S13.** The important habitat patches. (a)-(d) are the important habitat patches during different hydrology period
